# Magnetite Micro/Nanorobots for Efficient Targeted Alleviation of Inflammatory Bowel Disease

**DOI:** 10.1002/advs.202503307

**Published:** 2025-04-25

**Authors:** Ying Feng, Yang Liu, Linlin Liu, Qian Yang, Miao An, Huaming Yang

**Affiliations:** ^1^ Engineering Research Center of Nano‐Geomaterials of Ministry of Education China University of Geosciences Wuhan 430074 China; ^2^ Faculty of Materials Science and Chemistry China University of Geosciences Wuhan 430074 China; ^3^ Laboratory of Advanced Mineral Materials China University of Geosciences Wuhan 430074 China; ^4^ Centre for Immune‐oncology Nuffield Department of Medicine University of Oxford Old Road Campus Oxford OX3 7BN UK

**Keywords:** inflammatory bowel disease, intestinal milieu restoration, micro/nanorobots, resveratrol, ROS scavenger

## Abstract

Millions of people worldwide have inflammatory bowel disease (IBD). Self‐driven micro/nanorobots (MNRs) are efficient in the treatment of IBD. However, their lack of controllability regarding direction of motion in the organism and their inability to achieve continuous navigation limits their further application. In this study, polydopamine is wrapped around the magnetite surface, loaded with an anti‐inflammatory drug resveratrol, and wrapped with pH‐responsive sodium alginate to obtain magnetic MNRs. MNRs can be driven by magnetic fields to achieve directional movement and targeted transportation. In addition, MNRs can effectively remove reactive oxygen species from the inflammation site, repair intestinal damage, inhibit the cellular pathway of pro‐inflammatory factors, such as MAPK and NF‐κB pathways, and restore intestinal flora, thereby relieving IBDs. MNRs are safe and effective for in vivo treatment of IBD and have proven to be a promising therapeutic platform. This MNRs therapeutic strategy provides new insights into comprehensive IBD therapy.

## Graphical abstract

Improved reactive oxygen species scavenging capacities of functionalized micro/nanorobots (MNRs) are useful for the treatment of inflammatory bowel disease (IBD). MNRs can break the harmful bacteria in IBD, thereby eliminating inflammation and restoring the intestinal barrier function. With its excellent efficacy as an oral drug and superior biocompatibility, MNRs have great potential for clinical application in the treatment of IBD.

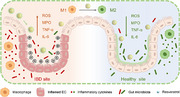

## 1 Introduction

Inflammatory bowel disease (IBD) is a chronic relapsing and remitting disease commonly categorized into: ulcerative colitis and Crohn's disease.^[^ ^1^ ^]^ The prevalence of IBD increased from 1990 to 2017, with huge implications for global health and the economy.^[^ ^2^ ^]^ IBD has a complex pathology characterized by elevated levels of inflammation, reactive oxygen species (ROS), disruption of the intestinal barrier, and abnormal intestinal flora.^[^ ^3^, ^4^, ^5^, ^6^ ^]^ This disease environment makes the treatment of IBD difficult. Currently, IBD is primarily treated with oral medications such as methotrexate (MTX)^[^ ^7^ ^]^ and 5‐amino salicylic acid (5‐ASA),^[^ ^8^ ^]^ that mitigate the inflammatory response. However, these drugs have limited effects on IBD. The amide group of MTX is easily hydrolyzed in acidic environments, making it difficult to maintain the activity of orally administered MTX.^[^ ^9^ ^]^ Intestinal metabolizing enzymes make 5‐ASA ineffective, leading to treatment failure.^[^ ^8^ ^]^ These drugs lack targeting mechanisms, which can easily cause adverse side effects and systemic adverse reactions.^[^ ^10^ ^]^ Additionally, imbalances in the gut microflora continue to induce inflammation in the intestinal area.^[^ ^11^ ^]^ If the medication does not change the imbalance in the intestinal flora, the anti‐inflammatory effect alone will only provide temporary relief.

Resveratrol (Res) is a polyphenol extracted from various natural species such as grapes, peanuts, and red wine. This substance acts as an antioxidant and maintains the functional integrity of the mucosal barrier. As a natural antioxidant, Res reduces ROS induced damage to mitochondria mainly by scavenging free radicals, reducing lipid peroxidation and modulating the activity of antioxidant‐related enzymes.^[^ ^12^ ^]^ Although Res has the potential to regulate intestinal flora,^[^ ^13^ ^]^ its poor water solubility, which results in low bioavailability, limits its use in organisms.^[^ ^14^ ^]^ To overcome this limitation, researchers have developed various nanocarriers including tetrahedral framework nucleic acids,^[^ ^12^ ^]^ hydrogels,^[^ ^15^, ^16^ ^]^ and polymeric micelles.^[^ ^17^ ^]^ These carriers improve the bioavailability of Res; however, they cannot actively propel it, especially in oral treatments that rely on biological fluids for passive transport. Thus, a long period of time is required for the drug to reach the lesion, which substantially reduces the timeliness of treatment. Therefore, it is crucial to design a drug delivery strategy that simultaneously relieves and precisely treats symptoms.

Micro/nanorobots (MNRs) are intelligent machines that can convert external energy into kinetic energy, and their driving methods are mainly categorized as self‐driven or driven by external power.^[^ ^18^, ^19^, ^20^, ^21^, ^22^, ^23^ ^]^ They are widely used for targeted drug delivery because they can actively travel through organisms to reach lesions that are difficult to reach using conventional drug delivery methods.^[^ ^24^ ^]^ Among these, self‐driven MNRs are often used for gastrointestinal drug delivery owing to the rich humoral environment in the gastrointestinal tract.^[^ ^25^, ^26^, ^27^ ^]^ Although self‐driven MNRs do not require additional equipment, they lack controllability with the direction of motion within an organism. Cao et al. reported using mesoporous manganese oxide (MnO_x_)‐based nanomotors driven by H_2_O_2_/ultrasound in a synergistic manner to cure colon cancer.^[^ ^28^ ^]^ These nanomotors do not target certain tissues or cells, nor are they able to move in a specific direction. Recently, magnetically driven MNRs have been recognized for their various advantages, such as precise and wireless three‐dimensional manipulation in physiological environments, access to small areas, and the use of harmless energy sources. They are promising tools for the precise delivery of drugs or cells to targeted areas^[^ ^29^ ^]^ and have been used to treat other diseases with good therapeutic results.^[^ ^30^, ^31^, ^32^ ^]^ Therefore, we propose the use of magnetite to prepare MNRs to improve their targeting ability for IBD treatment. Notably, to the best of our knowledge, this goal has not been achieved in the field of MNRs for IBD treatment.

**Figure 1:**
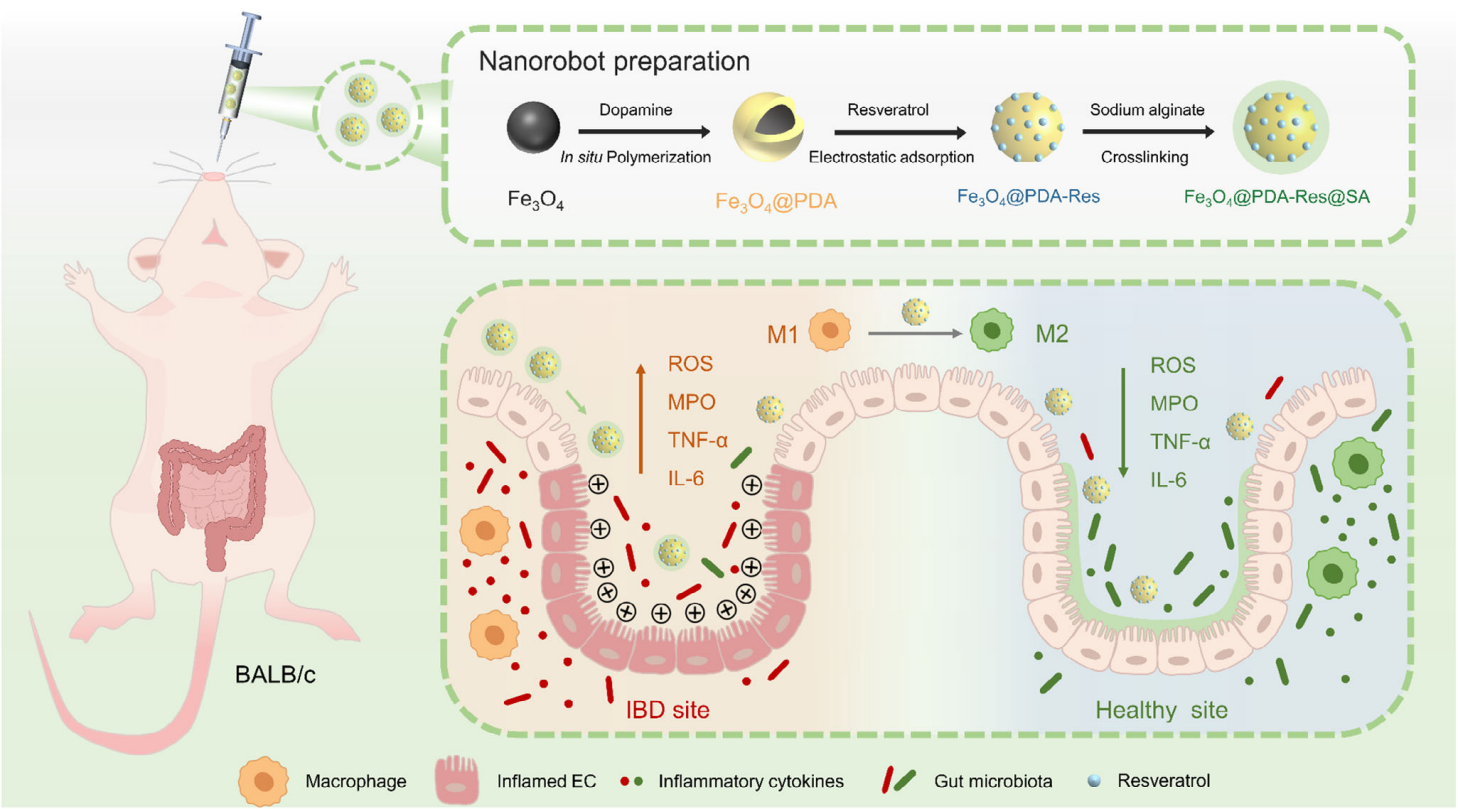
Schematic illustration of the preparation of MNRs and mechanisms of its activity in the treatment of IBDs. MNRs combines with positively charged intestinal inflammatory areas through electrostatic adsorption. They are able to remove reactive oxygen species, regulate intestinal flora disorders, repair damaged intestinal environments and alleviate intestinal diseases.

Here, we demonstrate that oral drug delivery for intestinal inflammation therapy can be achieved by safely and effectively using magnetic MNRs in vivo for colitis treatment. In this study, we prepared spherical magnetite as the main carrier of the MNRs. Magnetite spheres were wrapped in polydopamine (PDA) for electrostatic adsorption of Res and then coated with sodium alginate. They were simultaneously assembled under a magnetic field to form biocompatible MNRs with powerful thrust and precise navigation. After oral administration, the pH‐responsive sodium alginate shell protects the MNRs until they reach the gut. The negatively charged Fe_3_O_4_@PDA‐Res was then bound to the inflamed colon via electrostatic interactions (**Figure** **1**). MNRs were effective in repairing intestinal damage and restoring the intestinal environment in a mouse model induced by dextran sulfate sodium salt (DSS). Moreover, MNRs significantly inhibit ROS and inflammatory factor levels and ultimately restore the balance of gut bacteria; MNRs can effectively enhance their bioavailability. Overall, these MNRs have all the properties that require further development into a therapeutic tool for IBD. We believe that this study will provides new insights into the treatment of IBD.

## 2 Results and Discussion

### 2.1 Synthesis and Characterization of MNR

**Figure 2:**
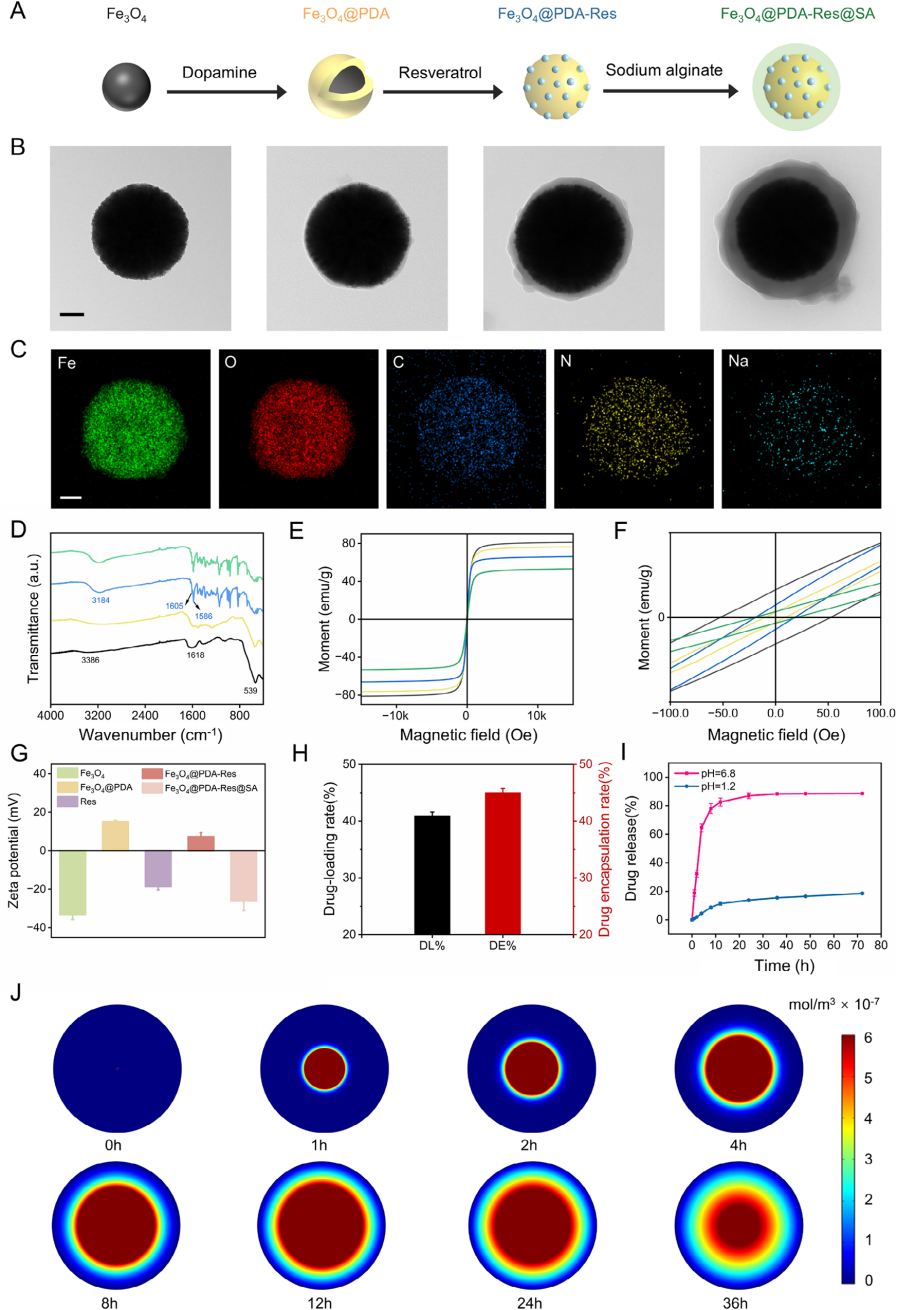
Fabrication and characterization of MNRs. A) Schematic of the fabrication process. B) TEM images of nanoparticles at each fabrication stage. Scale bar is 150 nm. C) EDX analysis of building block showing the elemental distribution of Fe, O, C, N and Na in a nanoparticle. Scale bar is 50 nm. D) FT‐IR spectra of nanoparticles at each fabrication stage, demonstrating the gradual change of surface functional groups. E) Magnetic hysteresis loop of nanoparticles at each fabrication stage. F) Localized magnification of the hysteresis loop around the origin (x range from −100 Oe to 100 Oe) of E). In (D)–(F), the black, yellow, blue, and green curves indicate Fe_3_O_4_, Fe_3_O_4_@PDA, Fe_3_O_4_@PDA‐Res, and Fe_3_O_4_@PDA‐Res@SA, respectively. G) Zeta potential evolution during preparation of Fe_3_O_4_@PDA‐Res@SA MNRs (*n* = 3; mean ± SD). H) Drug‐loading rate and drug encapsulation rate of Fe_3_O_4_@PDA‐Res@SA MNRs (*n* = 3; mean ± SD). I) Drug release under different pH conditions Fe_3_O_4_@PDA‐Res@SA MNRs (*n* = 3; mean ± SD). J) Simulation of drug release at pH 6.8.

MNRs were prepared using a convenient method and fully characterized to determine their nanostructures and modification progress. The preparation process consisted of four steps (**Figure** **2**A). First, Fe_3_O_4_ nanoparticles with a spherical structure were prepared using a solvothermal method.^[^ ^33^ ^]^ The nanoparticles were then dispersed in an alkaline buffer, followed by the addition of dopamine to form a PDA layer on their surfaces (Fe_3_O_4_@PDA) via an in situ polymerization process.^[^ ^34^ ^]^ Through electrostatic interactions, the negatively charged drug Res was adsorbed onto the polydopamine surface. Finally, sodium alginate (SA) was deposited on the surface to form a shell via a cross‐linking process. SA shells are biocompatible and pH‐responsive, stabilizing under acidic conditions in gastric juices and collapsing under neutral intestinal environments, resulting in on‐demand drug release without premature leakage. Transmission electron microscope (TEM) and scanning electron microscopy (SEM) images in Figure 2B and Figure S1 (Supporting Information) depict the morphology of the nanoparticles during each fabrication step. The synthesized Fe_3_O_4_ nanoparticles were homogeneous spheres, that uniformly formed thin surface layer after functionalization with PDA and SA. Energy dispersive X‐ray (EDX) analysis was performed to characterize the elements present in the resulting nanoparticles. The corresponding results in Figure 2C show that Fe and O are distributed over a very small area, whereas Na and C cover the entire area, indicating the presence of an SA shell layer. Additionally, N confirms the successful loading of PDA. The dynamic light scattering (DLS) results are shown in Figure S2 (Supporting Information). The average sizes of the Fe_3_O_4_, Fe_3_O_4_@PDA, Fe_3_O_4_@PDA‐Res, and Fe_3_O_4_@PDA‐Res@SA nanoparticles were ≈420, 500, 620, and 676 nm, respectively, indicating the gradual growth of the shell layer. The Fe_3_O_4_@PDA‐Res@SA nanoparticles were more stable in gastric fluid, and the SA shell layer was broken down in the intestinal fluid, resulting in a smaller size (Figure S3, Supporting Information). Fourier‐transform infrared (FT‐IR) spectroscopy was used to characterize the surface functional groups of the nanoparticles at each fabrication stage (Figure 2D). Iron oxide nanoparticles were successfully synthesized, as seen by the peaks at 539 (Fe‐O stretching vibration), 1618, and 3386 cm^−1^ (absorbed water and hydroxy groups) in all curves. The specific stretching vibration peaks of C = C and benzene ring skeleton at 1605 and  1586 cm^−1^ further confirmed the successful loading of Res. The magnetic properties were investigated using a vibrating sample magnetometer (VSM) at 300 K (Figure 2E). All nanoparticles were ferromagnetic, as shown by the hysteresis loops in Figure 2F. The gradually increasing proportion of nonmagnetic materials was the main cause of the saturation magnetization values decreasing from 82, 77, 66, and 53 emu g^−1^ for Fe_3_O_4_, Fe_3_O_4_@PDA, Fe_3_O_4_@PDA‐Res, and Fe_3_O_4_@PDA‐Res@SA, respectively. This suggests that Fe_3_O_4_@PDA‐Res@SA NPs have a strong magnetic attraction under an external magnetic field and can be used as components of clustered magnetic MNRs. Notably, the Fe_3_O_4_@PDA‐Res@SA NPs exhibit magneto thermal properties and can achieve temperature increases under magnetic fields (Figures S4–S8, Supporting Information). However, the temperature increase has little effect on the magnetic properties of the MNRs itself (Figure S9, Supporting Information).

When dispersed in an acidic solution at pH 2.0, the PDA layer exhibited a strong positive charge with a ζ‐potential of 15.10±0.65 mV (Figure 2G), thereby adsorbing the negatively charged drug Res through effective electrostatic self‐assembly. At a drug‐to‐carrier mass ratio of 10, the drug loading and encapsulation rates were ≈40.93% and 45.02%, respectively (Figure 2H; Figures S10 and S11, Supporting Information). To confirm the pH‐responsive Res release process from building block, solutions with pH value of 1.2 and 6.8 was used to simulate gastric and colonic conditions. At pH 1.2, the drug release rate was 18.56%, whereas upon immersion in pH 6.8 solution, they collapsed and released their contents at a drug release rate of 88.57% (Figure 2I). This suggest that SA has an ideal pH‐dependent disintegration behavior that protects the encapsulated Res from premature drug leakage in the harsh acidic gastric environment. In addition, the negative charge of Fe_3_O_4_@PDA‐Res@SA NPs is expected to promote their adhesion to positively charged inflamed colonic epithelial tissues. Finally, the drug release at pH 6.8 was simulated by software (Figure 2J).

### 2.2 Magnetic Propulsions of Magnetic MNRs

**Figure 3:**
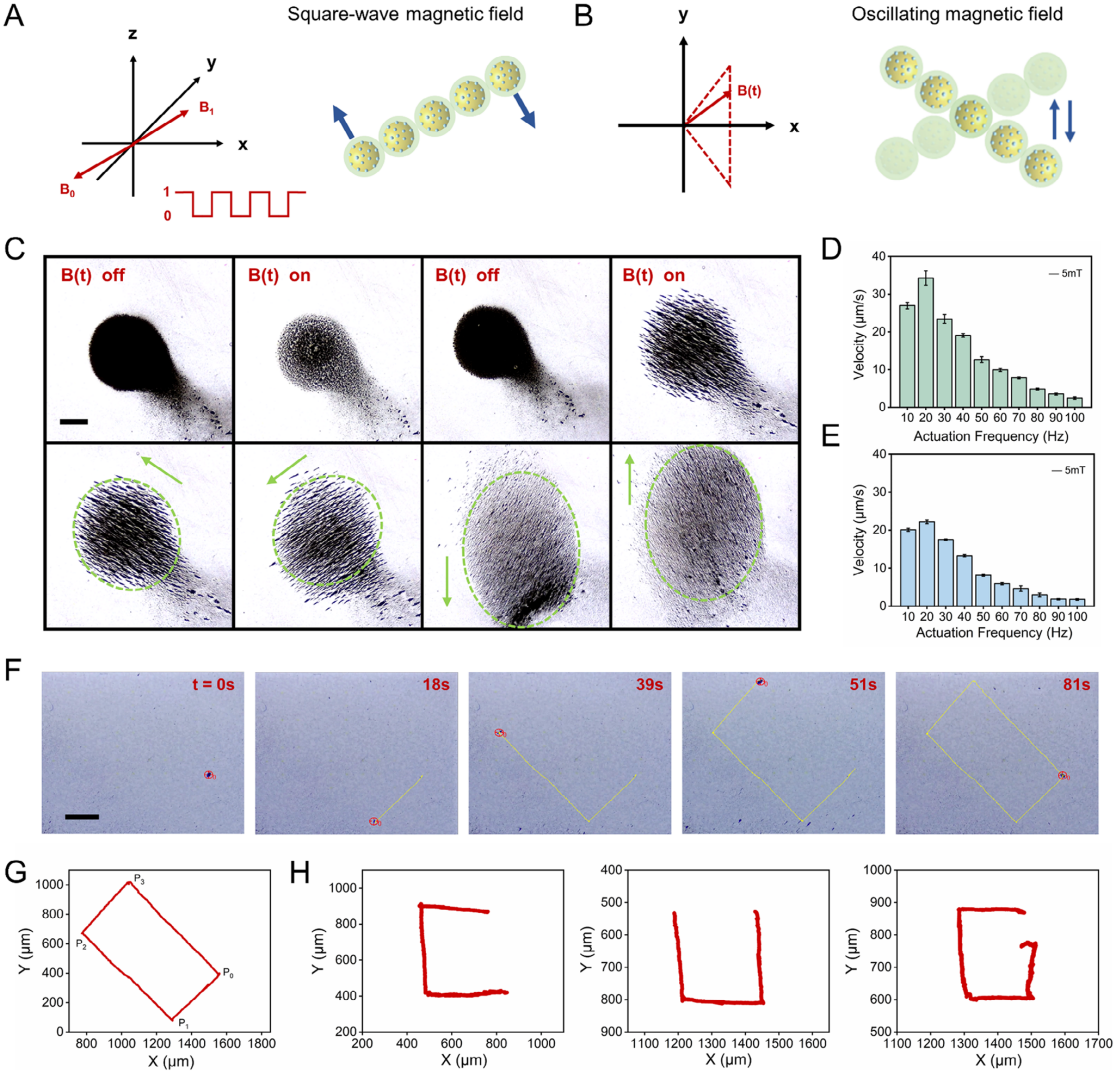
Motion performance of magnetic MNRs. A, B) Schematic illustration of the square‐wave magnetic field and oscillating magnetic field. The red and blue arrows represent the magnetic field and the direction of motion, respectively. C) MNRs undergo morphological changes and move in different directions under the control of a magnetic field (f = 5 Hz, 5mT). Scale bar: 2mm. D, E) Average velocity versus frequency in square‐wave magnetic field and oscillating magnetic field modes (*n* = 3; mean ± SD). F) Optical microscopy images of MNRs moving directionally to follow a rectangular trajectory (*f* = 5 Hz, 5mT). Scale bar: 2mm. G) Controllable navigation of MNRs along a rectangular route with a motion trajectory indicated by the red line (*f* = 5 Hz, 5mT). (H) Motion trajectory of the letter “CUG” indicated by the red line (*f* = 5 Hz, 5mT).

**Figure** **3**A,B provide a schematic representation of how the square‐wave and oscillating magnetic fields drive the behavior of the functionalized nanoparticles. A square‐wave magnetic field was applied using a magnetic field with square‐wave signals in the x, y, and z directions, where the amplitude and frequency of the magnetic field and the duty cycle of the square‐wave signals were adjustable. The oscillating magnetic field was created by applying a direct current signal in the x‐direction and sinusoidal signal in the y‐axis direction (Movie S1, Supporting Information). By adjusting the applied B(t) (Figure 3C), the MNRs were dynamically reconfigured, transforming from bulb‐like to dandelion‐like, dandelion‐like back to bulb‐like, and bulb‐like to ellipsoidal (Movie S2, Supporting Information). This suggests that the morphology of the MNRs is capable of reversible transitions. The direction of the MNRs can be controlled by adjusting B(t). The MNRs can move left‐up, left‐down, down and up in turn. When frequency is increased from 10 to 100 Hz, the MNRs in square magnetic field can move up to 34.29 µm s^−1^ (Figure 3D). The MNRs in oscillating magnetic field walk slower than those of the square magnetic field and exhibit a maximum velocity of 22.22 µm s^−1^ (Figure 3E). The relationship among the motion of the MNRs, magnetic field strength, and frequency was further explored. The driving frequency at which the maximum velocity was obtained was considered the critical value (critical frequency), beyond which the velocity decreased with increasing frequency. The motion of the MNRs follows this law when a fixed magnetic field strength of 3, 6, 9, and 12mT were applied to the square‐wave oscillating and oscillating magnetic field, respectively (Figure S12, Supporting Information). As the viscosity of the liquid environment increases, the velocity of the MNRs decreases. In addition, the velocities of the MNRs in simulated gastric fluid (SGF) and simulated intestinal fluid (SIF) were measured separately (Figure S13, Supporting Information). The slower velocities in gastrointestinal fluid may be due to the fact that gastric mucus and intestinal mucus give greater resistance to the movement of the MNRs. The MNRs could move in a predetermined rectangular and N‐shaped trajectory by adjusting the direction of B(t) (Figure 3F,G, Figure S14, Supporting Information), demonstrating their high‐performance motion controllability (Movie S3 and S4, Supporting Information). We control the MNRs move the trajectory of the letter “CUG” through a magnetic field (Figure 3H, Movies S5–S7, Supporting Information). Finally, we compared the performance of our MNR with that of other magnetically driven MNRs (Table S1, Supporting Information).

### 2.3 Anti‐inflammatory Effects In Vitro and Promote Intestinal Epithelial Barrier Repair

**Figure 4:**
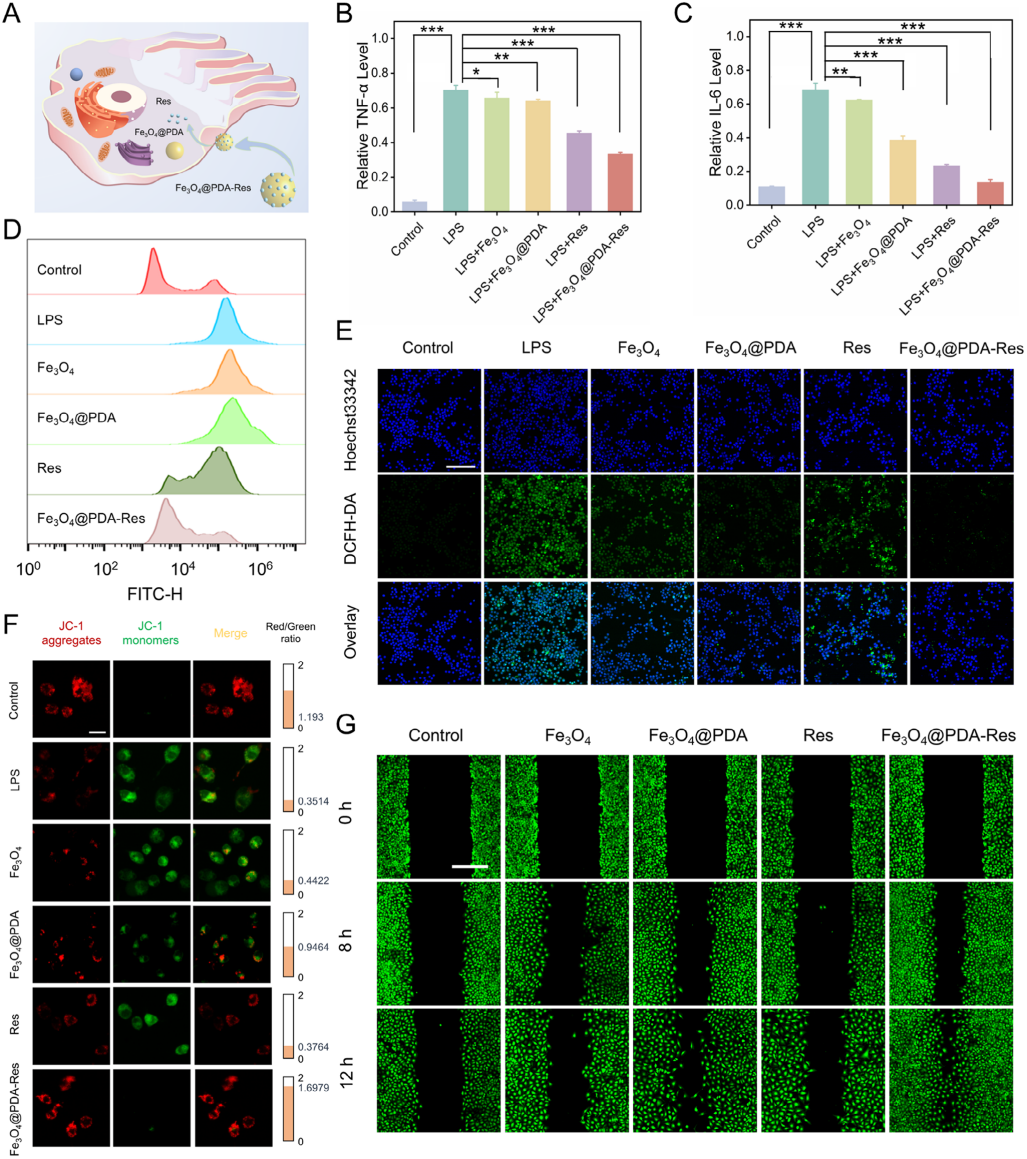
In vitro treatment of cells. A) Schematic diagram of Fe_3_O_4_@PDA‐Res releasing Res to anti‐inflammatory and promote proliferation and repair. The expression of B) IL‐6 and C) TNF‐α in the RAW264.7 cells under different treatment (Control, LPS, Fe_3_O_4_, Fe_3_O_4_@PDA, Res, and Fe_3_O_4_@PDA‐Res). D) ROS levels in untreated RAW264.7 cells and cells treated with four materials (Fe_3_O_4_, Fe_3_O_4_@PDA, Res, and Fe_3_O_4_@PDA‐Res) and stimulated by LPS. E) Representative ROS staining (green fluorescence) of RAW264.7 cells after various treatments. Scale bar: 100 µm. F) MMP monitored of RAW264.7 cells by staining with the JC‐1. Scale bar: 200µm. G) Scratch assay images of Caco‐2 cells cultivated in the medium supplementary with PBS (Control), Fe_3_O_4_, Fe_3_O_4_@PDA, Res, and Fe_3_O_4_@PDA‐Res. Scale bar: 300 µm. Data in (B–C) are shown as mean ± S.D, (*n* = 3 per group). (Statistical significance was performed by one‐way ANOVA test. **p < 0.01, ***p < 0.001, ****p < 0.0001, and ns: no significance).

The Fe_3_O_4_@PDA carrier and Res released by Fe_3_O_4_@PDA‐Res entered the cell for synergistic treatment (**Figure** **4**A). Lipopolysaccharide (LPS) was used to treat RAW264.7 macrophage cells increase the amount of intracellular ROS produced. Figure 4B,C show that IL‐6 and TNF‐α were significantly increased in LPS group compared with those in the healthy group, indicating that RAW264.7 cells were at a high level of inflammation. The Fe_3_O_4_@PDA‐Res treatment showed the best inhibitory effect on the above three inflammatory factors compared to that with Fe_3_O_4_, Fe_3_O_4_@PDA, and Res. IBD significantly increased release of several inflammatory factors (e.g., IL‐6 and TNF‐α) of NF‐κB. These pro‐inflammatory factors further increased ROS production, forming a vicious cycle of inflammation and ROS production. As shown in Figure 4D,E, Figures S15 and S16 (Supporting Information), Fe_3_O_4_@PDA‐Res significantly reduced intracellular ROS. The concentration of the material used in the cellular assay was determined from the results of the cytotoxicity assay (Figures S17 and S18, Supporting Information). Notably, the SA shell typically undergoes dissolution in the intestinal fluid; therefore, its interaction with cells has not been studied. The above evidence demonstrated that Fe_3_O_4_@PDA‐Res effectively eliminated ROS and pro‐inflammatory factors to break this vicious circle.

Homeostasis of epithelial cells depends on normal mitochondrial function;^[^ ^35^ ^]^ however, excessive ROS can damage the mitochondria and compromise epithelial cell activity.^[^ ^36^ ^]^ Thus, the JC‐1 probe was used to measure a change in the mitochondrial membrane potential (MMP). At higher or lower MMP, the probe forms a polymer or monomer in the matrix and emits a red or green fluorescent signal, respectively. The ratio of red to green fluorescence is an indicator of mitochondrial depolarization and early apoptosis.^[^ ^37^ ^]^ Figure 4F shows that control group cells exhibited a normal MMP, while RAW264.7, exposed to LPS, exhibited extensive green fluorescence, which was due to an increasing proportion of JC‐1 monomers and aggregates. Fe_3_O_4_@PDA‐Res completely restored MMP in the exposed cells. Caco‐2 cell migration is essential in intestinal barrier repair and determines the therapeutic effect in ulcerative colitis. In Figure 4G and Figure S19A (Supporting Information), Fe_3_O_4_@PDA‐Res shows a higher migration rate (88.62±0.41%) than that of Fe_3_O_4_ (30.23±1.72%), Fe_3_O_4_@PDA (59.66±0.79%), and Res (41.20±0.74%) at an interval of 12 h.

### 2.4 In Vivo Ulcerative Colitis Amelioration

**Figure 5:**
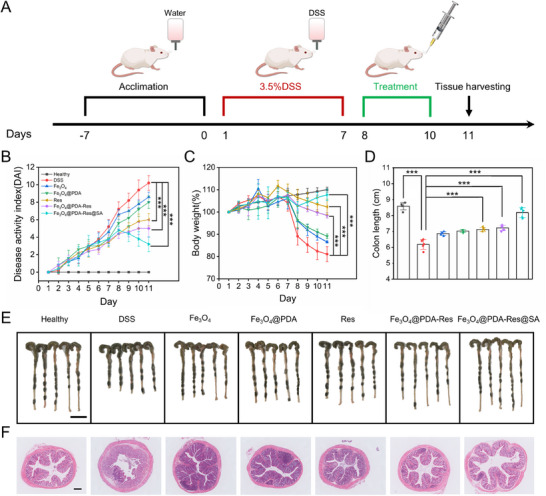
In vivo IBD amelioration. A) Schematic a description of the entire process of creating a model of colitis in mice generated by DSS and then administering oral therapies. B) DAI score values of the mice over 11 days of the experiment. C) Daily changes in mouse body weight determined during 11 experimental days. Colon length D) and digital photographs E) of the excised colons in the indicated groups. Scale bar: 2 cm. F) H&E‐stained colonic tissue sections from the indicated groups on day 11 of the experiment. Scale bar: 200 µm. Data in (B–D) are shown as mean ± S.D, (*n* = 5 per group). (Statistical significance was performed by one‐way ANOVA test. **p < 0.01, ***p < 0.001, ****p < 0.0001, and ns: no significance).

Owing to the excellent biocompatibility and ROS‐scavenging ability of Fe_3_O_4_@PDA‐Res, its in vivo therapeutic efficacy was further evaluated in a DSS‐induced BALB/C mouse IBD model (**Figure** **5**A). Enteritis was induced in mice after seven days of feeding with 3.5% DSS. The mice were randomly divided into seven groups: healthy (blank control group), enteritis (DSS, negative control group), Fe_3_O_4_ treatment (DSS+Fe_3_O_4_, experimental group), Fe_3_O_4_@PDA treatment (DSS+Fe_3_O_4_@PDA, experimental group), Res treatment (DSS+Res, experimental group), Fe_3_O_4_@PDA‐Res treatment (DSS+Fe_3_O_4_@PDA‐Res, experimental group) and Fe_3_O_4_@PDA‐Res@SA treatment (DSS+Fe_3_O_4_@PDA‐Res@SA, experimental group). All experimental groups received daily oral administration through gavage, and therapeutic efficacy was assessed by measuring the disease activity index (DAI), body weight, and colon length. Compared with the DSS group, the DSS+Fe_3_O_4_@PDA‐Res@SA group had a lower DAI (Figure 5B), higher body weight (Figure 5C), and longer colon length (Figure 5D,E). Compared with the group DSS+Res and healthy groups, there were fewer differences between the DSS+Fe_3_O_4_@PDA‐Res@SA and healthy groups. These results showed that Fe_3_O_4_@PDA‐Res@SA was more effective than Res in treating mice with DSS‐induced colitis. In the in vivo experiments, mice were orally administered Fe_3_O_4_@PDA‐Res@SA, and whole bowel fluorescence imaging 10 h later revealed a significant increase in Fe_3_O_4_@PDA‐Res@SA adhesion and retention in the mouse colon (Figure S19B, Supporting Information). Hematoxylin and eosin (H&E) staining of colon sections revealed that mice with colitis displayed significant crypt loss, histological collapse, substantial immune cell infiltration, and severe colonic epithelial damage in the inflamed colon. In contrast, the DSS+Fe_3_O_4_@PDA‐Res@SA group displayed an almost normal histological microstructure with minimal inflammatory cell infiltration (Figure 5F).

Notably, we compared its efficacy with that of the clinically available first‐line drugs, DEX (dexamethasone) and 5‐ASA. The results show that mice treated with DEX and 5‐ASA had DAI indices of 4.4 ± 0.55 and 3.6 ± 0.55, respectively, and colon lengths of 7.14 ± 0.22 cm and 7.36 ± 0.16 cm (Figure S20, Supporting Information). Therefore, MNRs showed superior therapeutic efficacy in two of the most important metrics of IBD (DAI and colon length) compared with that of other first‐line clinical agents. We used isolated pig intestinal tissues to mimic the mouse intestinal environment and achieved motility regulation of MNRs in the pig intestine (Figure S21; Movie S8, Supporting Information). It can cause morphological changes in and make full contact with the narrow intestinal wall to improve therapeutic efficiency (Movie S9 and S10, Supporting Information).

### 2.5 Anti‐Inflammation Activity In Vivo

**Figure 6:**
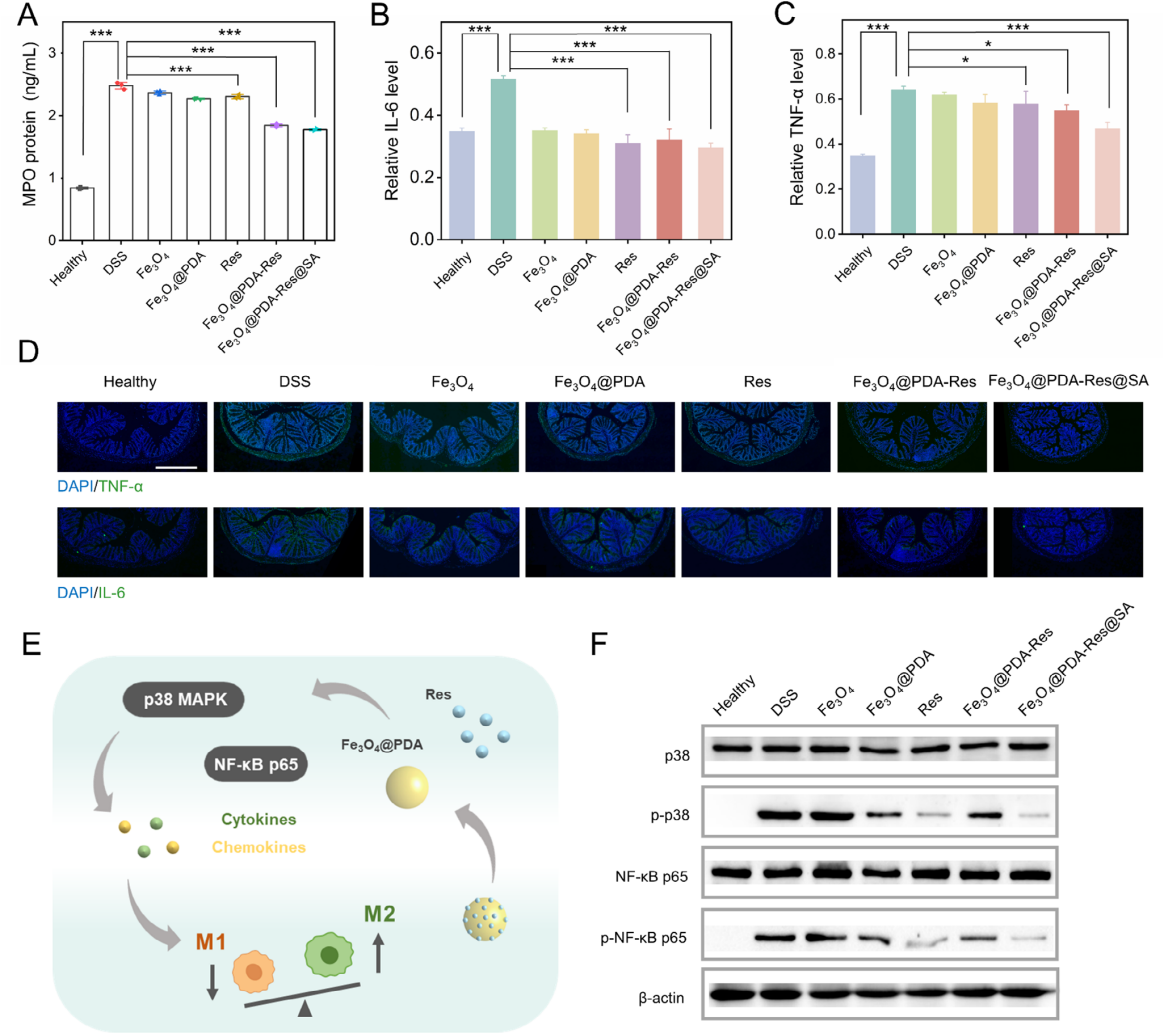
Anti‐inflammation activity in vivo. A) The amounts of MPO in colon issue excised from mice exposed to annotated treatments. Expression levels of inflammatory factors IL‐6 B) and TNF‐α C) in the serum of mice. D) Immunofluorescent staining images of TNF‐α/IL‐6 inflammatory factors in colon tissue section from different groups. Scale bar: 800 µm. E) Anti‐inflammatory by inhibition the activation of p38 MAPK and p65 NF‐κB. F) Western blot analysis showing the differential expression of p38, p‐p38, p65, and p‐p65 of different treatment groups in mice (healthy mice, IBD model mice, IBD treated with Fe_3_O_4_, IBD treated with Fe_3_O_4_@PDA, IBD treated with Res, IBD treated with Fe_3_O_4_@PDA‐Res, and IBD treated with Fe_3_O_4_@PDA‐Res@SA. Data in (A–C) are shown as mean ± S.D, (*n* = 3 per group). (Statistical significance was performed by one‐way ANOVA test. **p < 0.01, ***p < 0.001, ****p < 0.0001, and ns: no significance).

Neutrophils express myeloperoxidase (MPO), which regulates IBD by catalyzing the synthesis of reactive chemicals and hypochlorous acid in inflamed tissues.^[^ ^38^ ^]^ **Figure** **6**A suggests that although the MPO levels were elevated in the colon tissue of the DSS group, Fe_3_O_4_@PDA‐Res@SA treatment significantly reduced their aggregation, demonstrating that Fe_3_O_4_@PDA‐Res@SA could effectively relieve the symptoms of IBD. The results presented in Figure 6B–D demonstrate a considerable increase in TNF‐α and IL‐6 in IBD lesions compared with those in the healthy group. This suggests that the intestinal tissues of IBD mice were highly inflamed. The Fe_3_O_4_@PDA‐Res@SA treatment showed the best inhibitory effect on the above three inflammatory factors compared to that with Fe_3_O_4_, Fe_3_O_4_@PDA, Res, and Fe_3_O_4_@PDA‐Res. MAPK and NF‐κB signaling pathways are activated in colon tissues of patients with IBD and DSS‐induced colitis mice.^[^ ^39^ ^]^ Therefore, the potential effects of Fe_3_O_4_@PDA‐Res@SA intake on the activation of p38 MAPK and NF‐κB p65 signaling pathways were explored (Figure 6E,F). The results showed that NF‐κB p65 and p38 MAPK signaling pathways were activated in the colon tissues of DSS‐induced colitis mice. Fe_3_O_4_@PDA‐Res@SA intake reduced the phosphorylation of NF‐κB p65 and p38 MAPK, thus exhibiting anti‐inflammatory effects.

### 2.6 Therapeutic Mechanisms of MNRs on IBD

**Figure 7:**
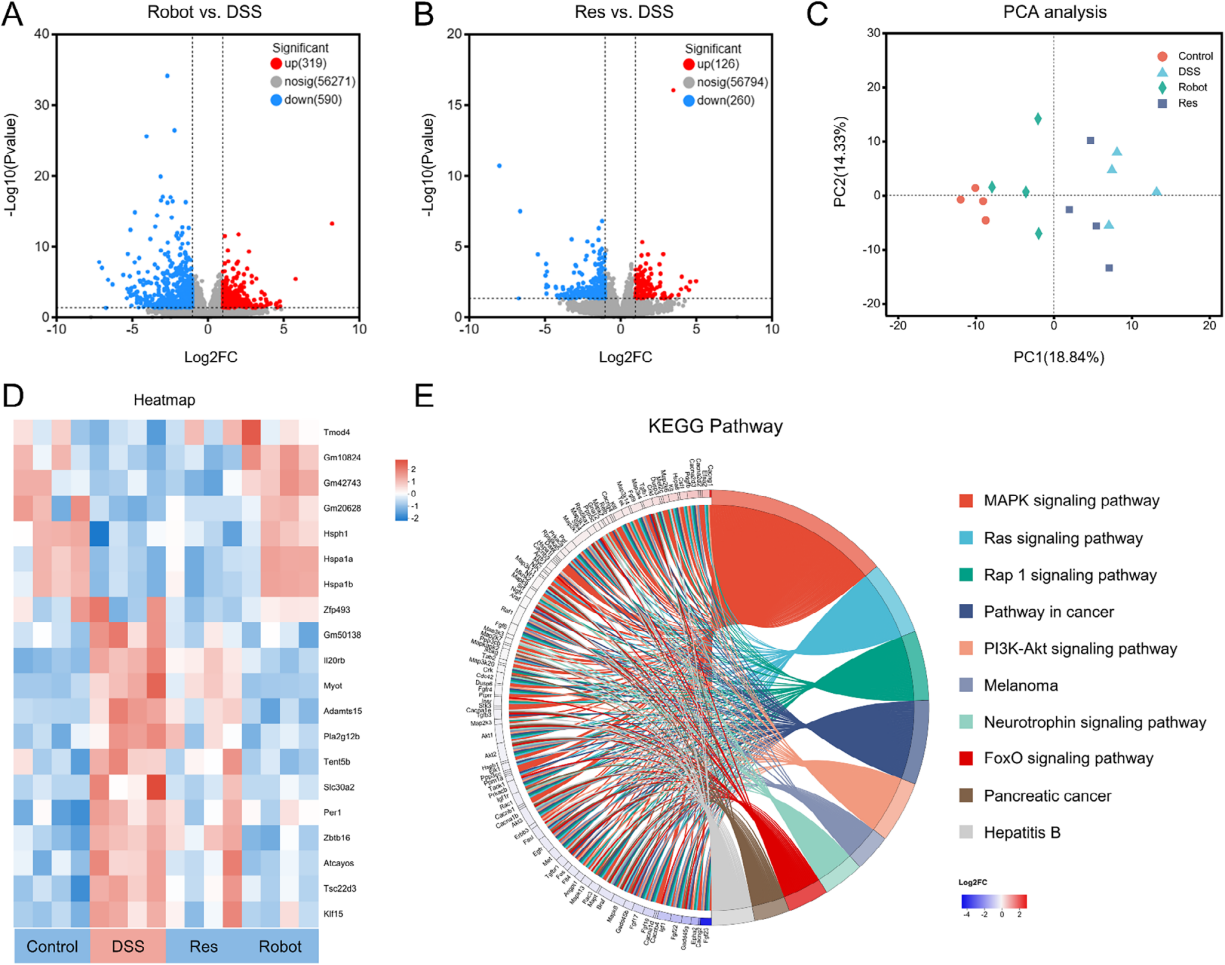
Transcriptomic analysis. A)Volcano plot of differentially expressed genes determined between DSS+Robot and DSS group. B) Volcano plot of differentially expressed genes determined between DSS+Res and DSS group. C) The principal components analysis of sample from each group. D) Heat map of RNA‐seq analysis showing the genes in the healthy, DSS, DSS+Res, and DSS+Robot groups (fold change in right). E) KEGG pathways enrichment analysis of ROS‐related signaling pathway in DSS versus DSS+Robot.

To elucidate the therapeutic mechanism of MNRs in IBD, RNA‐seq was performed on the mouse colonic tissue. RNA‐seq of colon tissues from the Robot‐treated, Res‐treated, DSS, and healthy groups showed that these groups had different transcriptome profiles. After comparing Robot‐treated colitis animals to DSS mice, the volcano plot revealed that 319 genes were up‐regulated and 590 genes were downregulated, indicating a significant change in gene expression (**Figure** **7**A). The Robot group showed higher variability in all, upregulated, and downregulated genes than the Res group (Figure 7B). These results suggest that the therapy was effective. Principal component analysis further confirmed this finding (Figure 7C). Subsequent investigation of the normalized heatmap (which shows the top 20 differentially expressed genes) indicated that the colitis animals treated with the robot were more similar to the healthy group in the gene profile (Figure 7D).

Screened genes were subjected to enrichment and clustering analyses to clarify their underlying therapeutic processes. Kyoto Encyclopedia of Genes and Genomes (KEGG) and Gene Ontology (GO) enrichment analyses (Figures S22 and S23, Supporting Information) were performed to investigate the biological roles of these DEGs and main enrichment pathways in more detail. Functional clustering analysis of samples in ROS‐related signaling pathways was performed using the KEGG database, with log2 (fold change) as the evaluation criterion. The findings showed that 10 KEGG pathway categories—MAPK, Ras, Rap1, cancer, PI3K‐Akt, Melanoma, Neurotrophin, FoxO, Pancreatic cancer, Hepatitis B signaling pathways—were linked to variations in gene expression between the DSS and DSS+Robot groups (Figure 7E, Figure S24, Supporting Information). Previous studies have shown that by reducing ROS levels, and inhibiting NF‐κB and other inflammatory signaling pathways, robots decrease colon inflammation in mouse models. The inflammatory signaling pathway regulates the release of inflammatory factors from cells, and the binding of inflammatory factors to receptors further activates the inflammatory signaling pathway, forming a positive feedback loop that exacerbates the inflammatory response.^[^ ^40^ ^]^ The results of differential gene analysis showed enrichment in the MAPK signaling pathway, indicating that MNRs significantly reduced the expression of inflammatory factors, TNF‐α and IL‐6, by blocking this loop, further enhancing the anti‐inflammatory effect. Notably, robot treatment altered the PI3K‐Akt signaling pathway, indicating that it may play a role in regulating intestinal epithelial barrier repair and intestinal microbiota in IBD treatment.

Short‐chain fatty acids (SCFAs), products of the fermentation of undigested polysaccharides by anaerobic bacteria in the gut, ameliorate the inflammatory response by modulating immune cells and enhancing intestinal barrier integrity.^[^ ^41^, ^42^ ^]^ The development of colitis in mice significantly reduces the diversity of the intestinal flora, leading to a decrease in SCFA production and worsening of the disease.^[^ ^43^ ^]^ We performed KEGG enrichment analysis on several key genes involved in SCFA metabolism (e.g., acetic, and propionic acid metabolism), suggesting that our therapeutic regimen could address this issue to some extent (Figure S25, Supporting Information).

### 2.7 Restore Gut Microbiota Mechanism of MNRs on IBD

**Figure 8:**
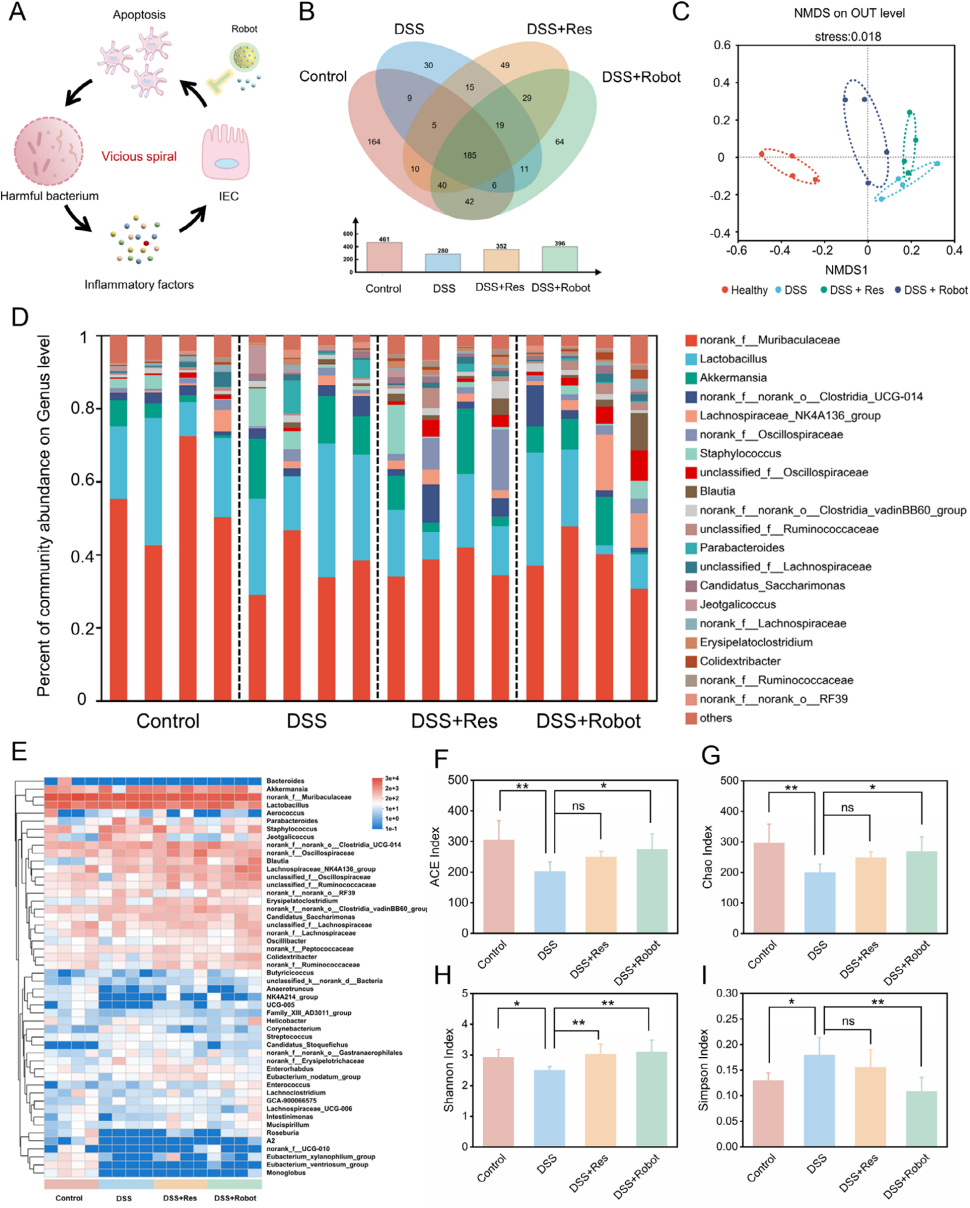
The effect of MNRs on the gut microbiota of IBD mice. A) Schematic diagram of MNRs breaking the vicious cycle of intestinal epithelial cell apoptosis and large‐scale bacterial colonization to induce inflammation. B) Venn diagram of the intestinal bacterial species detected in the control, DSS, DSS group exposed to Res, and DSS group exposed to Robot. C) NMDS analysis of the microbiota composition. D) Bar chart of the percent of community abundance of gut microbiota in the healthy group, DSS model group, Res treatment group, and Robot treatment group. E) Heatmap showing the relative abundance of gut microbiota composition analysis at the order level in each group. F–I) Microbial community richness and diversity analyses of normal and treated mice expressed as ACE F), Chao G), Shannon H) and Simpson I) indices. Data in (F–I) are shown as mean ± S.D, (*n* = 4 per group). (Statistical significance was performed by one‐way ANOVA test. **p < 0.01, ***p < 0.001, ****p < 0.0001, and ns: no significance).

There was a considerable difference in the intestinal flora of IBD lesions compared with that in healthy intestines. In IBD, harmful bacteria infiltrate the intestinal wall and excessive ROS disrupt the integrity of the intestinal mucus and epithelial barrier, triggering an intense inflammatory response that leads to intestinal epithelial cell (IEC) apoptosis, creating a vicious cycle between IEC apoptosis and colonization by dangerous microorganisms (**Figure** **8**A).^[^ ^44^, ^45^ ^]^ In this regard, ROS act as inflammatory mediators that activate toll‐like receptors, thereby inducing the release of inflammatory cytokines.^[^ ^46^, ^47^ ^]^ This process leads to the release of large amounts of inflammatory cytokines through plasma membrane rupture, resulting in cell death and accelerating the progression of inflammation.^[^ ^48^, ^49^ ^]^ Apoptosis provides key nutrients to the IBD pathogenic flora, leading to colonization of pathogenic bacteria and resulting in an imbalance of the intestinal flora.^[^ ^44^ ^]^ MNRs are able to break this cycle by scavenging ROS and inhibiting the expression of cellular inflammatory factor. Compared with the DSS group, the Venn diagram showed that MNR treatment increased the number of intestinal microbes at the operational taxonomic unit (OTU) level (Figure 8B). Nonmetric multidimensional scaling (NMDS) analysis of the weighted UniFrac distance was performed to show the degree of similarity in the microbial composition between the groups. The Robot‐treated group showed a different phylogenetic architectural profile compared with that of the IBD group (Figure 8C). Notably, the mice treated with MNRs had microbiomes more similar to those of healthy control mice. In addition, nanorobot treatment increased the relative abundance of beneficial bacteria (norank_f_Muribaculaceae) in IBD lesions, whereas Res treatment was less effective than MNRs (Figure S26, Supporting Information). These results suggest that MNRs inhibit harmful bacteria and increase beneficial bacteria, ultimately restoring the intestinal flora to a healthy balance.

The relative abundance at the class level is shown in Figure 8D. The gut microbiota of the different groups was mainly composed of Bacteroidetes, Bacilli, Clostridia, Verrucomicrobiae, and Saccharimonadia. The top 50 strains with the highest abundance were further analyzed at the species level. The heatmap presented in Figure 8E provides additional evidence that the microbiota species composition of the DSS group underwent significant changes, whereas the microbiota of mice treated with MNRs had a composition similar to that of healthy mice. The acquired data verified that MNRs could alter the microbial composition profile of IBD mice from dysbiosis to homeostasis. Oral MNR treatment improved the community richness expressed by OTU, ACE index (Figure 8F), Chao index (Figure 8G), community diversity Shannon index (Figure 8H), and Simpson index (Figure 8I). As shown in the previously described investigations, MNRs effectively altered the gastrointestinal milieu by eliminating ROS and altering the gut microbiota.

### 2.8 Biocompatibility of MNRs

**Figure 9:**
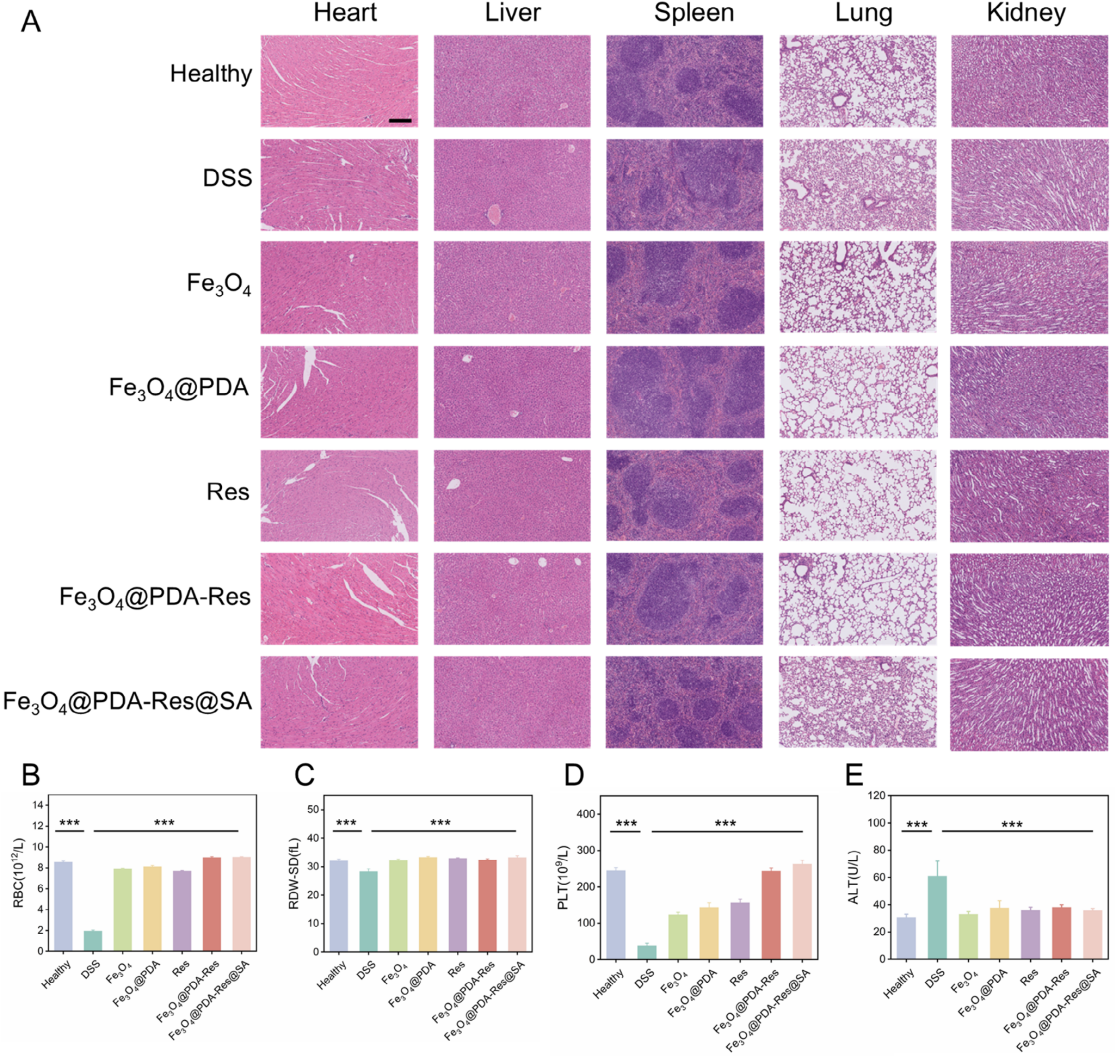
In vivo biosafety assessment. A) H&E‐stained histological sections of major organs excised from mice after annotated treatments. Scale bar: 200 µm. B) RBC indicators, C) RDW‐SD indicators, D) PLT indicators and E) ALT indicators of each group. Data in (B–E) are shown as mean ± S.D, (*n* = 3 per group). (Statistical significance was performed by one‐way ANOVA test. **p < 0.01, ***p < 0.001, ****p < 0.0001, and ns: no significance).

Finally, to confirm that the clinical transformation of MNRs is feasible, the safety and biocompatibility of the treatment were assessed. No hazardous components were detected in the MNRs. Even after treating RAW264.7 cells in vitro for 24 h at escalating MNRs doses (from 0 to 1280 µg mL^−1^), we did not notice a discernible decline in their viability (Figurse S17 and S18, Supporting Information). This demonstrated the high degree of cellular biocompatibility of MNRs. Oral MNRs were collected only from the gastrointestinal tract, therefore, there was no risk to critical organs (**Figure** **9**A). As anticipated, there were no indications of systemic toxicity, autoimmunity, or disease in the main organs during MNR therapy. After oral administration of MNR, liver function (alanine aminotransferase [ALT], lactic dehydrogenase [LDH]), and renal function (creatinine [CR]), blood index parameters (red blood cells [RBC], mean platelets [PLT], and red cell distribution width‐SD [RDW‐SD]) were not affected (Figure 9B–E; Figure S27, Supporting Information). To assess the potential in vivo safety of magnetothermal therapy, brain tissues from mice undergoing magnetothermal therapy were collected for H&E staining. Despite the fact that the abdominal temperature of the mice rose to nearly 50 °C under alternating magnetic field, no significant brain damage was detected (Figure S28, Supporting Information). The therapeutic effect of MNRs minimized damage to other organs. Its metabolism in organisms has been studied using pharmacokinetics; it is metabolized and eliminated from the body relatively quickly (Figure S29, Table S2, Supporting Information). ICP‐MS was used to evaluate the biosafety and metabolism of the MNRs. Mice treated with MNRs did not exhibit elevated levels of metal ions in their heart, liver, spleen, lung, or kidney (Figure S30, Supporting Information). This evidence suggests that MNRs have excellent biocompatibility with potential for clinical translation.

## 3 Discussion

IBD is a chronic and recurrent inflammatory disease of the gastrointestinal tract that substantially increases the risk of colon cancer.^[^ ^50^ ^]^ The pathogenesis of IBD is complex and clinicopathological studies have shown that intestinal flora dysbiosis is closely associated with the development of IBD.^[^ ^51^ ^]^ The traditional clinical approach to treatment has been primarily through the administration of anti‐inflammatory drugs to patients with IBD; however, these drugs have targeting issues that can lead to systemic side effects and serious complications.^[^ ^52^ ^]^ Intestinal inflammation leads to a dramatic increase in ROS level in the intestinal mucosa and most current research has focused on the topical delivery of antioxidants to achieve anti‐inflammatory therapy in IBD.^[^ ^53^, ^54^ ^]^ However, restoring intestinal ecological balance and repairing the epithelium barrier cannot be accomplished by solely treating inflammation without also considering the dysbiosis of the gut flora. Therefore, a drug carrier that effectively scavenges ROS and enables the modulation of the intestinal flora is urgently needed. MNRs exhibit unique propulsive forces in liquid media and can be targeted by delivering drugs directly to the disease site.^[^ ^55^, ^56^, ^57^, ^58^, ^59^ ^]^ In particular, chemically driven MNRs are commonly used to treat gastrointestinal disorders and use biological fluid responses for locomotion.^[^ ^25^ ^]^ Their speed and direction cannot be controlled.

In this study, we constructed biocompatible MNRs that can reversibly change their morphology under the control of a magnetic field and achieve directed motion. The carboxyl group (‐COOH) of SA is protonated at low pH in the gastric acidic environment and intermolecular hydrogen bonding is enhanced to form a dense structure without drug release; under alkaline conditions, the carboxyl group is deprotonated (–COO–), the molecule is negatively charged, and electrostatic repulsion leads to gel dissolution and solubilization. Thus, SA shells exhibit pH‐responsive behavior and are capable of releasing drugs in the gastrointestinal environment. However, passive navigation, which relies on humoral transport, results in slow and poorly targeted drug delivery and reduces therapeutic efficiency. With the introduction of magnetic navigation, MNRs achieved controllable speeds and directionality. Therefore, the integration of the pH responsiveness of SA shells with magnetic navigation can substantially enhance the delivery efficiency and therapeutic effect of drug carriers. In addition, the material synthesis process was reproducible and the structure and properties of the samples from each batch were consistent, confirming the stability and reliability of the synthesis method. A magnetic field device for the modulation of MNRs, which can be combined with clinical imaging devices (e.g., endoscopes), may enable a wider range of biomedical MNRs applications for clinical therapy. Overall, MNRs in this study have the ability to scavenge ROS, repair wounds, and regulate flora, showing potential for the treatment of various inflammatory diseases, such as gastrointestinal inflammation, diabetic wounds, and atherosclerosis.

## 4 Conclusion

In summary, we demonstrated the significant potential of MNRs as an oral therapy for IBD. Functionalized MNRs exhibit enhanced ROS‐scavenging capabilities for the diagnosis and treatment of IBD. The ability of MNRs to efficiently target IBD in vivo has demonstrated a highly effective therapeutic effect against IBD, which is significantly better than that of Res. The therapeutic mechanism of MNRs has been profoundly and systematically revealed, mainly by inhibiting the p38 MAPK and p65 NF‐κB signaling pathway. MNRs can break the harmful bacteria in IBD, thereby eliminating inflammation and restoring the intestinal barrier function. Given the advantages of MNR as an oral therapy, with strong efficacy and superior biocompatibility, they have substantial potential for clinical translation for IBD treatment. Furthermore, the viability of Res as a small‐molecule medication for in vivo transformation is increased by the MNR design, which can influence the creation of nano‐drug carriers for further small‐molecule medications.

## 5 Experimental Section

### Physicochemical Characterization Tools and Approaches

TEM images were obtained with a field emission transmission electron microscope (JEOL JEM‐2100F, Japan). Hydrodynamic diameters and zeta potentials were measured on zeta potential and particle size analyzer (Brookhaven 90Plus PALS, USA). Absorption spectra were acquired using microplate reader measurement (Tecan Spark, Austria). FT‐IR spectra were obtained using an infrared spectrophotometer (Thermo Fisher Nicolet iS50, USA). Hysteresis loops were gained with a vibrating sample magnetometer (LakeShore7404, USA). CLSM images were captured with a Leica STELLARIS 5 microscope (Leica, Germany). Quantification of fluorescence intensity of cells using flow cytometry (BD Accuri C6 plus, USA).

### Preparation of Fe_3_O_4_ Nanoparticles

First, 1.35 g of FeCl_3_∙6H_2_O was dissolved in 40 mL of EG by magnetic stirring to form an orange‐yellow transparent solution, and then 3.6 g of NaAc and 1 g of PEG were added. A yellow‐brown turbid mixture was obtained after 4 h of vigorous stirring. Subsequently, the mixture was sealed in a 50 mL reactor and heated at 200 °C for 10 h before cooling naturally to room temperature. The black product was collected with a permanent magnet and washed three times with deionized water to remove the residual solvent. Finally, the nanoparticles were vacuum dried at 60 °C for 12 h and collected for storage.

### Preparation of Fe_3_O_4_@PDA Nanoparticles

A smooth PDA layer was encapsulated on the Fe_3_O_4_ surface by in situ polymerization.^[^ ^60^ ^]^ Dissolve 0.12 g Tris in 100 mL deionized water and disperse by sonication. The pH of the resulting solution was adjusted to 8.5 with 1 M hydrochloric acid to form an alkaline buffer solution. The buffer solution was poured into a 250 mL beaker and 2 mL of Fe_3_O_4_ nanoparticle solution (50 mg mL^−1^) was added. Then, the mixture was stirred for 30 min to completely disperse the magnetite nanoparticles. Subsequently, 0.02 g of dopamine hydrochloride was added to the mixture and was mechanically stirred for 4 h at room temperature to make the polymerization reaction complete. Finally, the mixture was collected with permanent magnets, washed three times with deionized water and dried under vacuum at 60 °C for 12 h.

### Preparation of Fe_3_O_4_@PDA‐Res Nanoparticles

25 mg of resveratrol powder was weighed and dispersed in 20 mL of methanol, and ultrasonically dispersed for 5 min. 2.5 mg of Fe_3_O_4_@PDA was then taken and continued to be ultrasonically dispersed for 5 min, after which the mixture was shaken away from the light at 37 °C for 24 h. Finally, the product was collected with a magnet, washed with methanol for three times, and freeze‐dried to obtain the resveratrol‐containing magnetite complexes Fe_3_O_4_@PDA‐Res.

Using UV spectrophotometry, a standard concentration curve of Res was initially created in order to calculate the drug loading of Fe_3_O_4_@PDA. 200 µL of supernatant from the drug loading process was collected, and the UV absorption spectrum of the supernatant was measured. Using the standard curve, the concentration of Fe_3_O_4_@PDA in the supernatant was determined. The formulas for drug encapsulation efficiency and loading efficiency are as follows:

(1) $$\mathrm{Encapsulation}\;\mathrm{efficiency}\;\left(\%\right)=\frac{W_{\mathrm{Loaded}\;\mathrm{Res}}}{W_{Total\;Res}}\times 100\%$$

(2) $$\mathrm{Loading}\;\mathrm{efficiency}\;\left(\%\right)=\frac{W_{\mathrm{Loaded}\;\mathrm{Res}}}{W_{Fe_{3}O_{4}@PDA-Res}}\times 100\%$$

### Preparation of Fe_3_O_4_@PDA‐Res@SA Nanoparticles

The oil phase was prepared as follows: 57.2 mL of liquid paraffin was taken in a round‐bottomed flask, heated to 50 °C in a water bath, 1.2 mL of Span‐80 and 0.4 mL of Tween‐80 were added, and the mixture was mechanically stirred until it was transparent and clarified and then cooled to 37 °C. The aqueous phase was prepared as follows: 12 mg of Fe_3_O_4_@PDA‐Res prepared and 20 mg of sodium alginate were dispersed in 2 mL of deionized water and mechanically stirred for 30 min. The aqueous phase was added dropwise into the oil phase at a rate of 500 µL min^−1^ using a syringe pump, maintaining a temperature of 37 °C and a rotational speed of 700 rpm during the process. When the oil phase is fully mixed with the water phase, continue mechanical stirring for 1 h to make a uniform and stable milky white water‐in‐oil emulsion. Add 5% calcium chloride solution drop by drop for crosslinking, mechanical stirring and curing for 2 h, respectively, washed with isopropyl alcohol and deionized water for 3 times. After freeze‐drying, the Fe_3_O_4_@PDA‐Res@SA nanoparticles were obtained.

### pH‐Responsive Drug Release

In order to simulate the drug release in the gastrointestinal environment, Fe_3_O_4_@PDA‐Res@SA nanoparticles were immersed in artificial gastric fluid at pH 1.2 and artificial intestinal fluid at 6.8. The cumulative drug release rate was calculated over time.

### Drug Release Simulation

The phenomenon of drug diffusion by MNRs within the intestinal fluid was computationally simulated using the dilute matter transfer module of COMSOL Multiphysics software. The concentration of MNRs was maximum in the center region and zero in the rest of the region at t = 0.

### ABTS•+ Scavenging Assay

The antioxidant capacity of the materials was assessed by using the total antioxidant capacity assay kit (Beyotime, China). The ABTS solution was mixed 1:1 with the oxidizing agent to configure an ABTS workhorse solution, which was stored at room temperature and protected from light for 12 h. The ABTS workhorse solution was diluted with 80% ethanol until the absorbance was about 1.4. The solution was then incubated with a range of materials in the dark for 10 min. The mixture was centrifuged at 8000 rpm for 5 min and the absorbance of the supernatant at 405 nm was measured using microplate reader.

### Magnetothermal Effect

A high‐frequency induction heating device (Shuangping SPG‐10AB‐II, China) supplied the alternating magnetic field (AMF). The material was placed in a centrifuge tube at the center of the coil and heated, and the temperature changes were recorded by an infrared thermographic camera (Fotric 326, China).

### Magnetic Propulsions of MNRs

We built our own experimental setup for the magnetic propulsions of the MNRs (Figure S31, Supporting Information). The magnetic actuation experiments were conducted in a customized three‐axis Helmholtz electromagnetic coil setup fixed on an operating microscope (RWD, China). At first, the suspension of the Fe_3_O_4_@PDA‐Res@SA NPs was added into a glass‐bottom tank and placed in the working space of the electromagnetic coils. Then, square‐wave magnetic field and oscillating magnetic field were applied by the electromagnetic coils that were controlled. The magnetic propulsion of the Fe_3_O_4_@PDA‐Res@SA NPs were observed and recorded through the operating microscope. The speed of Fe_3_O_4_@PDA‐Res@SA NPs were analyzed using the Video Spot Tracker V08.01.02 software.

### Cell Culture

RAW264.7 cells, Human colon carcinoma cells (Caco‐2) were cultured in minimum essential medium supplemented with 20% fetal bovine serum, 1% nonessential amino acid, 1% sodium pyruvate, 1% GlutaMAX, 1% l‐glutamine, and 1% penicillin/streptomycin. The cells were maintained at 37 °C with 5% CO_2_.

### ROS Scavenging In Vitro

RAW264.7 cells were seeded in a six‐well plate and cultured in a CO_2_ incubator at 37 °C until an appropriate cell density was reached. Fe_3_O_4_ (20 µg mL^−1^), Fe_3_O_4_@PDA (20 µg mL^−1^), Res (20 µg mL^−1^), and Fe_3_O_4_@PDA‐Res (50 µg mL^−1^) were added to the RAW264.7 cell culture dishes along with LPS (2 µg mL^−1^). The cells were incubated in the treatment conditions for 3 h. 2′,7′‐dichlorodihydrofluorescein diacetate (DCFH‐DA, 0.5 µg mL^−1^) probe was added and incubated at 37 °C in the CO_2_ incubator for 50 min. Subsequently, the cells were washed thrice with a serum‐free culture medium. The collected cells were resuspended in PBS and analyzed for intracellular ROS levels using flow cytometry and laser scanning confocal microscope.

### Cytotoxicity Evaluation of MNRs

RAW264.7 cells were seed in 95‐well plates at a density of 2 × 10^5^ of cells per well for 24 h. The cells were then incubated with various concentrations of Fe_3_O_4_, Fe_3_O_4_@PDA, Fe_3_O_4_@PDA‐Res, Res, and Fe_3_O_4_@PDA‐Res@SA (0, 5, 10, 20, 40, 80, 160, 320, 640, 1280 µg mL^−1^) for 12 or 24 h. Then, the medium was removed and cell viability was determined by the CCK‐8 method. Briefly, 0.1 m of CCK‐8 solution was added to each well and incubated at 37 °C for 1 h. Relative cell viability was finalized by measuring the absorbance of each well at 450 nm using an enzyme marker.

### Mitochondrial Membrane Potential Measurements

RAW264.7 cells were seeded in 6‐well plates at a density of 1 × 10^4^ of cells per well. Fe_3_O_4_ (20 µg mL^−1^), Fe_3_O_4_@PDA (20 µg mL^−1^), Res (20 µg mL^−1^), and Fe_3_O_4_@PDA‐Res (50 µg mL^−1^) were added to the RAW264.7 cell culture dishes along with LPS (2 µg mL^−1^) for 3 h. Afterward, the cells were rinsed with PBS and stained in the dark with JC‐1 (1 µg mL^−1^) at 37 °C for 30 min. Microscopy images were captured using CLSM.

### In Vitro Wound Healing Properties

The wound healing properties of different NPs were evaluated using the scratch method. Caco‐2 cells are a human epithelial cell line used in the intestinal epithelial barrier model. Cells were inoculated in 6‐well plates at 1 × 10^6^ cells per well. After 24 h of incubation, the monolayers were scored with a pipette tip, rinsed with PBS, and incubated before and after the addition of Fe_3_O_4_ (20 µg mL^−1^), Fe_3_O_4_@PDA (20 µg mL^−1^), Res (20 µg mL^−1^), and Fe_3_O_4_@PDA‐Res (50 µg mL^−1^) suspension to image the “wound”. The unhealed areas of the NPs were measured using ImageJ software to evaluate their wound healing ability.

### Enzyme‐Linked Immunosorbent Assay (ELISA) Analysis

To evaluate the levels of inflammatory factors, enzyme‐linked immunosorbent assay (ELISA) was used to determine the levels of TNF‐α, IL‐6 and MPO in cell supernatants, mouse serum and mouse colon homogenates. Mouse intestinal tissue homogenates were prepared at 4 °C. Each sample was centrifuged at 10 000 × *g* for 10 min at 4 °C. The levels of TNF‐α, IL‐6 and MPO enzyme activity in the supernatant were determined by ELISA.

### DSS‐Induced Model of IBD

Female BALB/C mice (20g , 6–8 weeks) were cohoused for 7 days. Afterward, mice were randomly assigned to the set groups with 5 mice per group. The mice were normally fed and drinking water was set as the control group, while the other groups were treated with drinking water containing 3.5% DSS for 7 days. Then, DSS‐induced mice were orally administered different treatments (Fe_3_O_4_, Fe_3_O_4_@PDA, Res, Fe_3_O_4_@PDA‐Res, Fe_3_O_4_@PDA‐Res@SA) from day 8 to day 10. During the experimental period, the body weight change and DAI of the mice were recorded daily. The DAI scoring criterion were (for a total of 0–10 points): i) weight loss (no change = 0 points, 0–5% = 1 point, 6–10% = 2 points, 11–20% = 3 points, >20% = 4 points); ii) stool properties (normal = 0 points; soft, intact granular shape = 1 point; soft, unshaped stool = 2 points; diarrhea = 3 points); and iii) bleeding (none = 0 points, presence of blood = 2 point, visible bleeding = 4 points). All groups of mice were sacrificed on day 11 for subsequent procedures, including excision of the entire colon and major organs for length measurements, H&E‐stained, western blot analysis and biosafety estimation. All animal experimental procedures involved in this study were approved by the Institutional Animal Welfare and Ethics Committee of Jarvis (Wuhan) biological pharmaceutical Co.,Ltd (JWS‐20240123‐007).

### Microbiome Analysis

A mouse fecal sample was taken and DNA extraction was performed. After extracting the total DNA of the samples, primers were designed based on conserved regions and sequencing adapters were added at the end of the primers. The products were PCR amplified, purified, quantified and normalized to construct a sequencing library. Sequencing was performed using Illumina, and the PE reads obtained from sequencing were firstly spliced according to overlap relationship, while the sequence quality was quality controlled and filtered.

### Biocompatibility and Biodistribution

H&E‐stained colonic tissue sections were used for histological assessment. The blood and major organs (colon, heart, liver, lung, kidney and spleen) of the sacrificed mice were collected for histopathological analysis. Each organ was fixed with paraformaldehyde and embedded in paraffin. Tissues were stained with H&E and photographed by microscopy. Routine blood and blood chemistry analyses to assess systemic toxicity. In addition, ICP of iron ions in various organs of mice was analyzed three days after oral injection to assess biological metabolism.

### Pharmacokinetics

Mice were weighed, and the Fe_3_O_4_@PDA‐Res@SA nanoparticles were given by gavage at a dose of 50 mg kg^−1^. Blood was collected from the retro‐orbital venous plexus of mice 0.083, 0.25, 0.5, 1, 2, 4, 8, and 12 h after the administration of the drug, and was placed in 1.5 mL EP tubes containing EDTA, centrifuged at 5000 r min^−1^ for 15 min, plasma samples were separated, and stored at −80 °C for measurement. The plasma concentrations at each time point were processed by DAS2.0 software, and the pharmacokinetic parameters of resveratrol were calculated according to the nonatrial model.

### Statistical Analysis

All results are presented as the means ± SD. The significance between the two groups was analyzed by an unpaired two‐tailed Student's t test. For multiple comparisons, a one‐way analysis of variance (ANOVA) with Tukey's post hoc test was used. P values of less than 0.05 were considered significant. *P < 0.05, **P < 0.01 and ***P < 0.001.

## Conflict of Interest

The authors declare no conflict of interest.

## Author Contributions

H.Y. developed the concept for the work. Y.F. and H.Y. conceived the project and designed the experiments. Y.F. performed the experiments and characterized the samples, and wrote the draft of the manuscript. Y.L. performed the numerical simulation. L.L. and M.A. discussed the results and interpreted the data. Q.Y. proposed amendments. H.Y. wrote the final manuscript and supervised the research. All authors approved the final version of the manuscript.

## Supporting information



Supporting Information

Supplemental Movie 1

Supplemental Movie 2

Supplemental Movie 3

Supplemental Movie 4

Supplemental Movie 5

Supplemental Movie 6

Supplemental Movie 7

Supplemental Movie 8

Supplemental Movie 9

Supplemental Movie 10

## Data Availability

The data that support the findings of this study are available from the corresponding author upon reasonable request.;
