# Supplementary material for: Magnetite Micro/Nanorobots for Efficient Targeted Alleviation of Inflammatory Bowel Disease
Source: Adv Sci (Weinh). 2025 Apr 25;12(26):2503307. doi: 10.1002/advs.202503307 (PMC12245070; doi:10.1002/advs.202503307)
Supplement: Supplementary file 1 — Supporting Information [file ADVS-12-2503307-s006.docx]

Supplementary Materials for

**Magnetite Micro/nanorobots for Efficient** **Targeted Alleviation of Inflammatory Bowel Disease**

Ying Feng *et al.*

*Corresponding author: Huaming Yang. Email: [hm.yang@cug.edu.cn](mailto:hm.yang@cug.edu.cn;)

**This file includes:**

Figs. S1 to S31

**Other Supplementary Materials for this manuscript include the following:**

Table S1 to S2

Movies S1 to S10


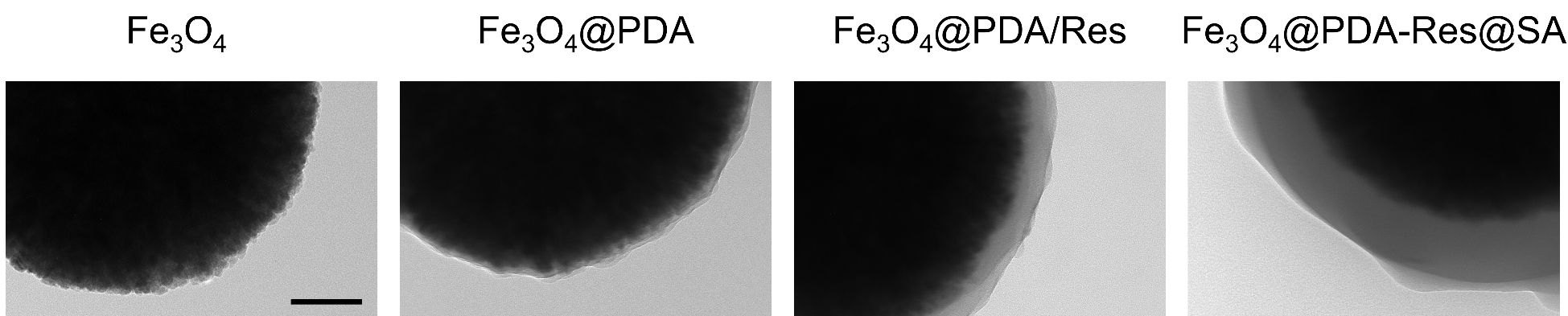


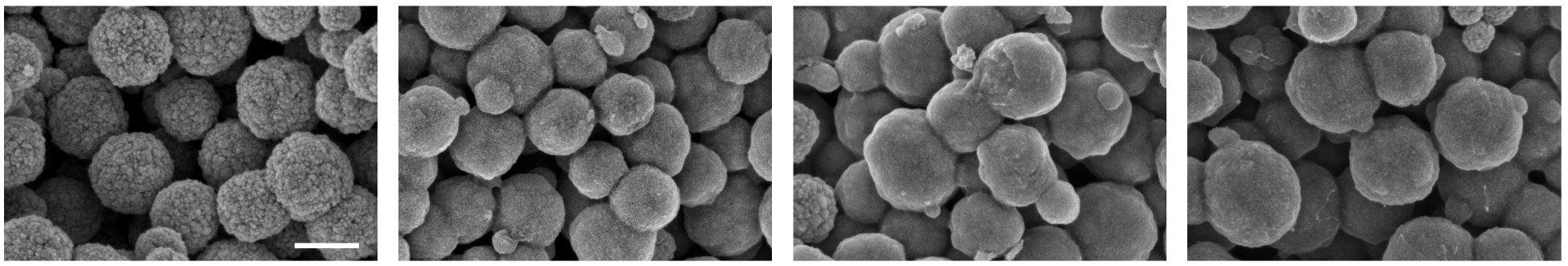


Figure S1. Local TEM and SEM images of nanoparticles at each fabrication stage. Scale bar is 100 nm and 200 nm.


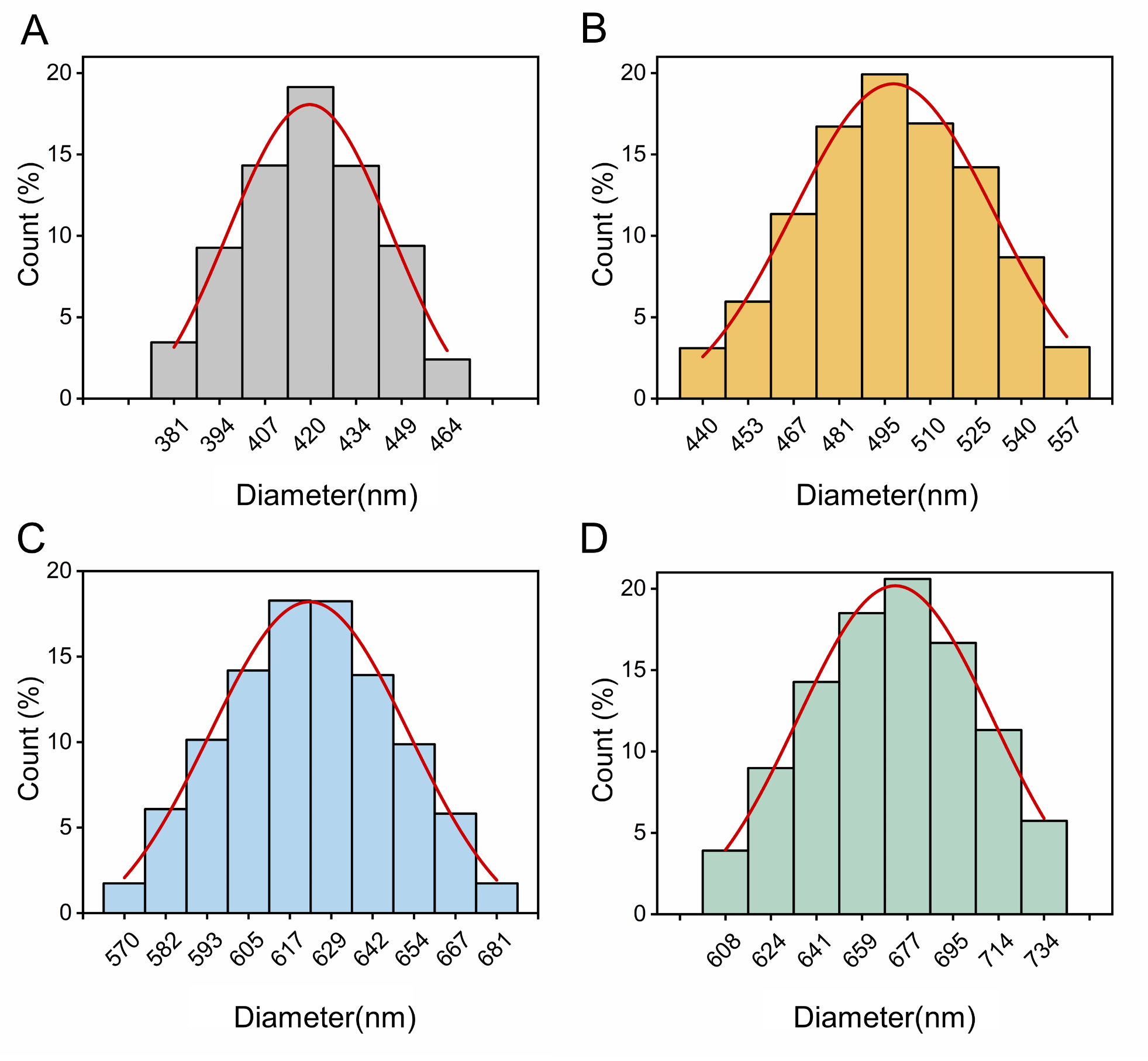


Figure S2. Corresponding diameter distributions of nanoparticles at each fabrication stage. (A) Fe_3_O_4_, (B) Fe_3_O_4_@PDA, (C) Fe_3_O_4_@PDA-Res, (D) Fe_3_O_4_@PDA-Res@SA.


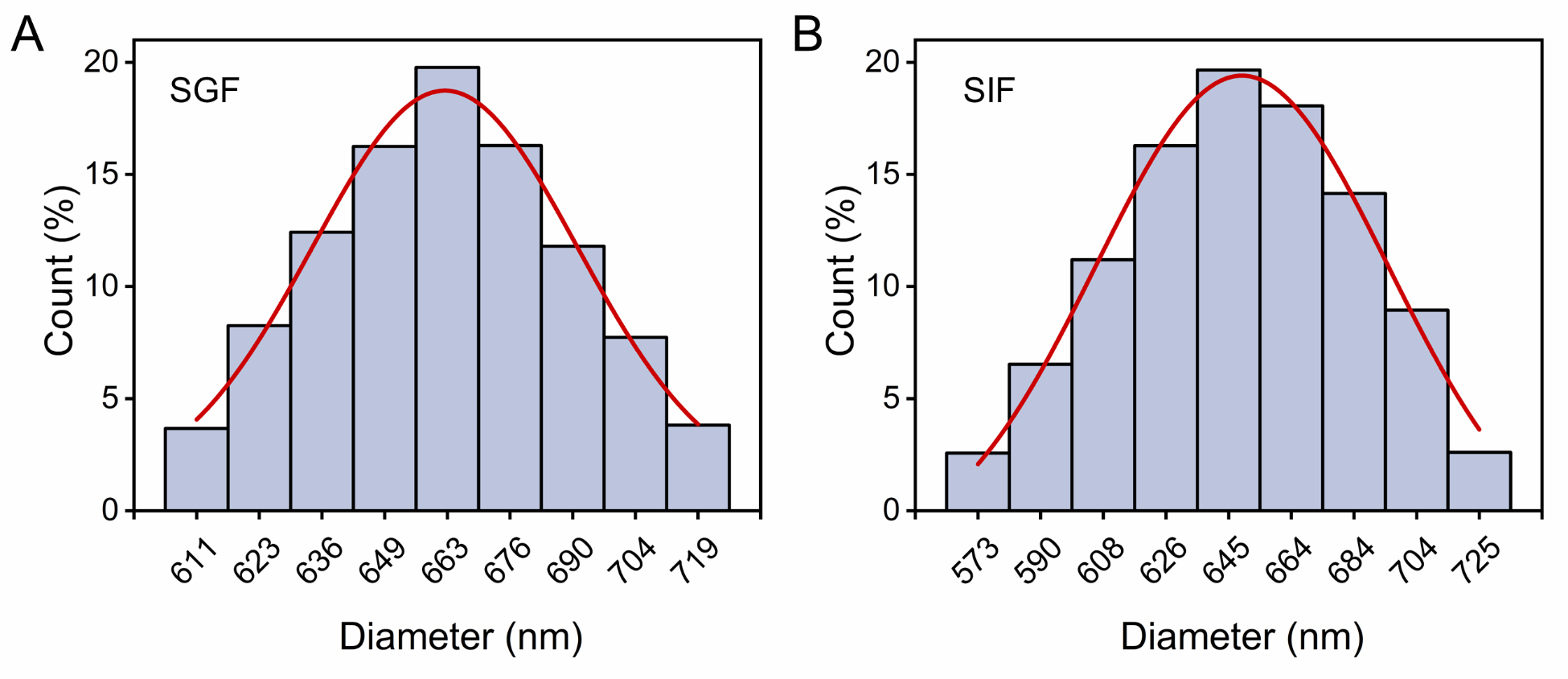


Figure S3. Corresponding diameter distributions of Fe_3_O_4_@PDA-Res@SA after 2h in SGF solution and SIF solution, respectively. SGF: Simulated Gastric Fluid, SIF: Simulated Intestinal Fluid.


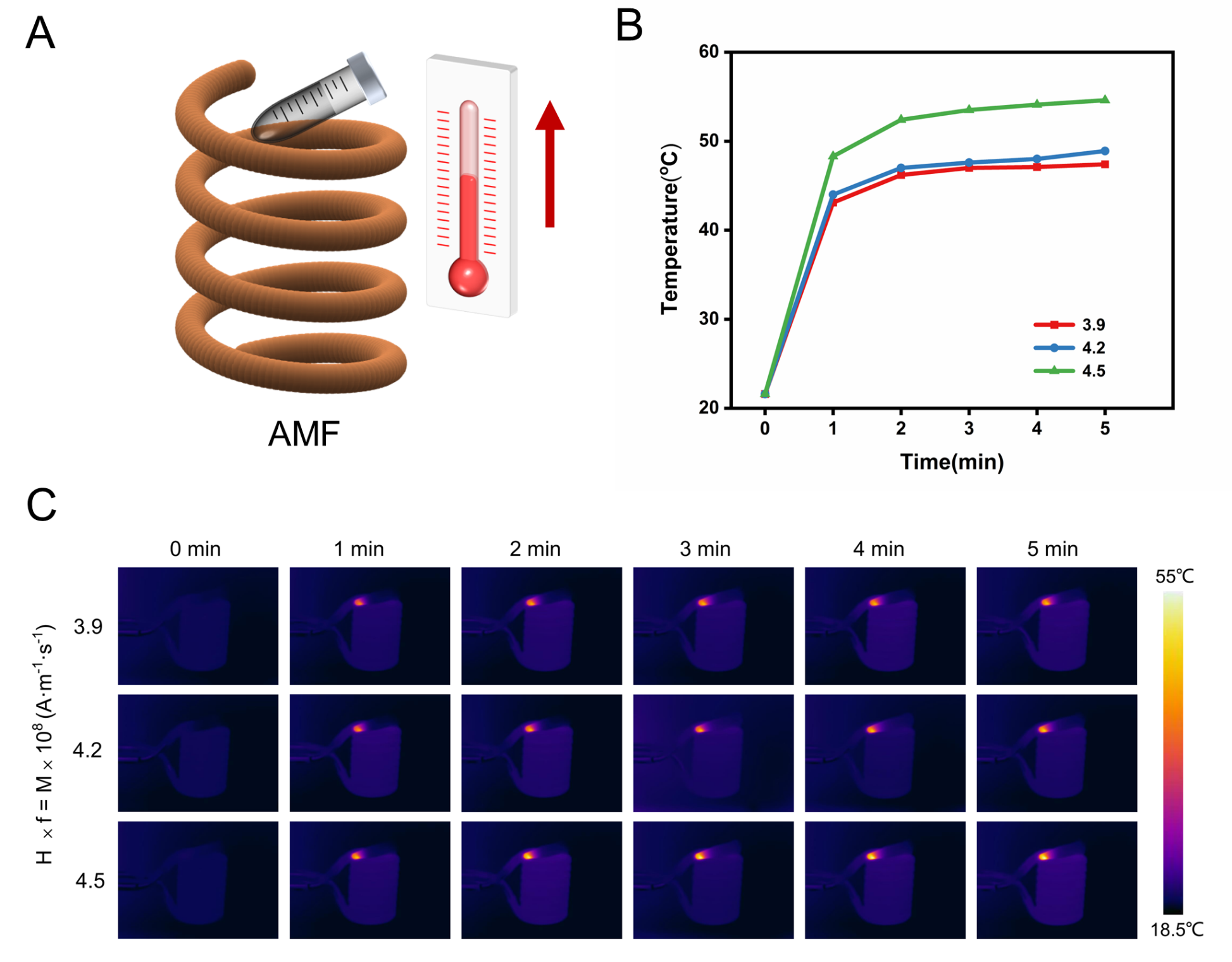


Figure S4. Magneto-thermal effect of MNRs. (A) Schematic diagram of a magnetic coil heating nanoparticles. (B) Temperature change curves of Fe_3_O_4_@PDA-Res@SA NPs under different field intensities (H × f = M × 10^8^ A·m^−1^·s^−1^; M = 3.9, 4.2, and 4.5). (C) Magnetothermal heating/cooling curves of Fe_3_O_4_@PDA-Res@SA (2mg mL^-1^) after four cycles of alternating magnetic field. (D) Infrared thermal images of Fe_3_O_4_@PDA-Res@SA NPs under different field intensities (H × f = M × 10^8^ A·m^−1^·s^−1^; M = 3.9, 4.2, and 4.5).


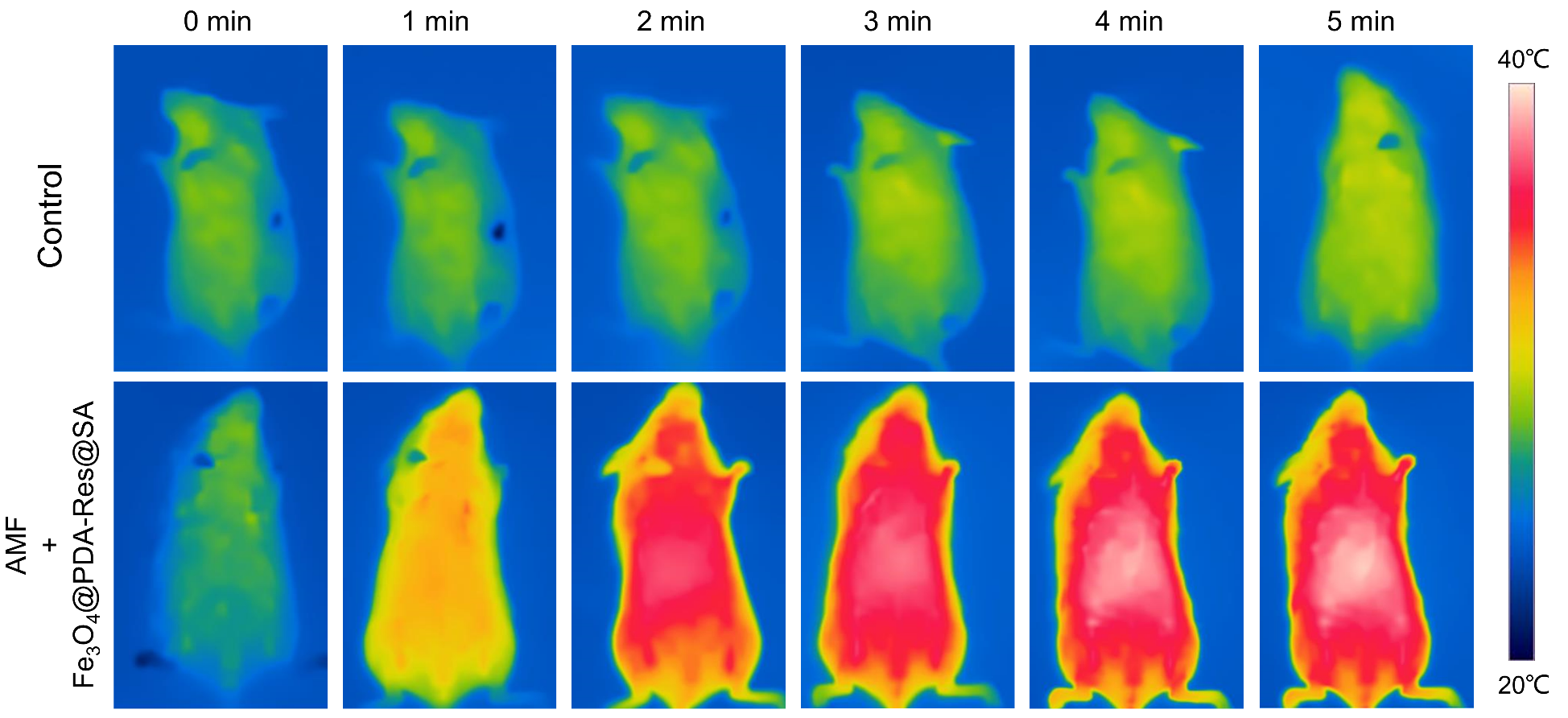


Figure S5. Infrared thermal images of the control and Fe_3_O_4_@PDA-Res@SA+AMF groups under AMF for 5 min in vivo.





Figure S6. Temperature rise of magnetic nanoparticles.


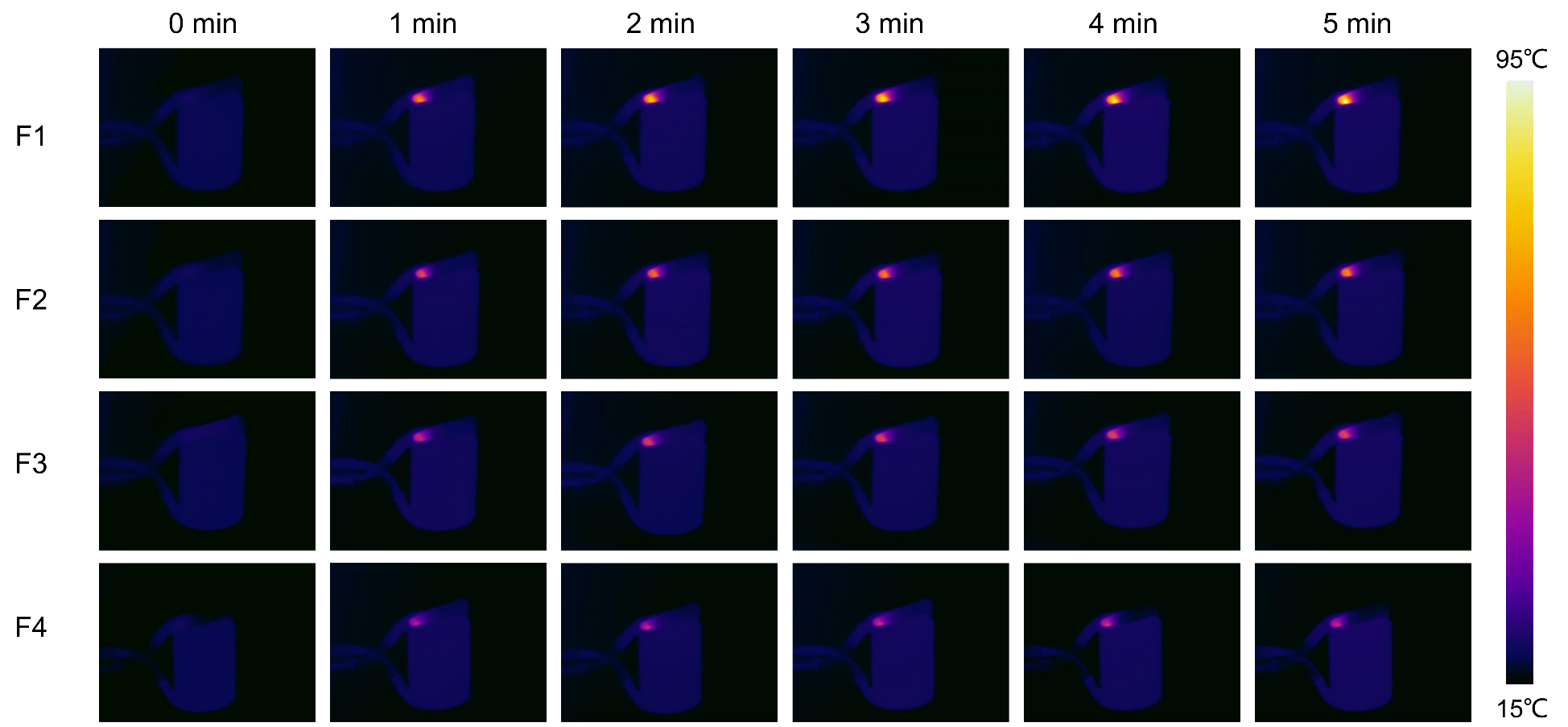


Figure S7. Infrared thermal images of magnetic nanoparticles (F1=Fe_3_O_4_, F2=Fe_3_O_4_@PDA, F3=Fe_3_O_4_@PDA-Res, F4=Fe_3_O_4_@PDA-Res@SA NPs) under the same AMF intensity.





Figure S8. Temperature change curves of the control and Fe_3_O_4_@PDA-Res@SA+AMF groups under AMF for 5 min in vivo.





Figure S9. Variation of magnetic properties of Fe_3_O_4_@PDA-Res@SA NPs with temperature.





Figure S10. Drug standard curve for resveratrol.


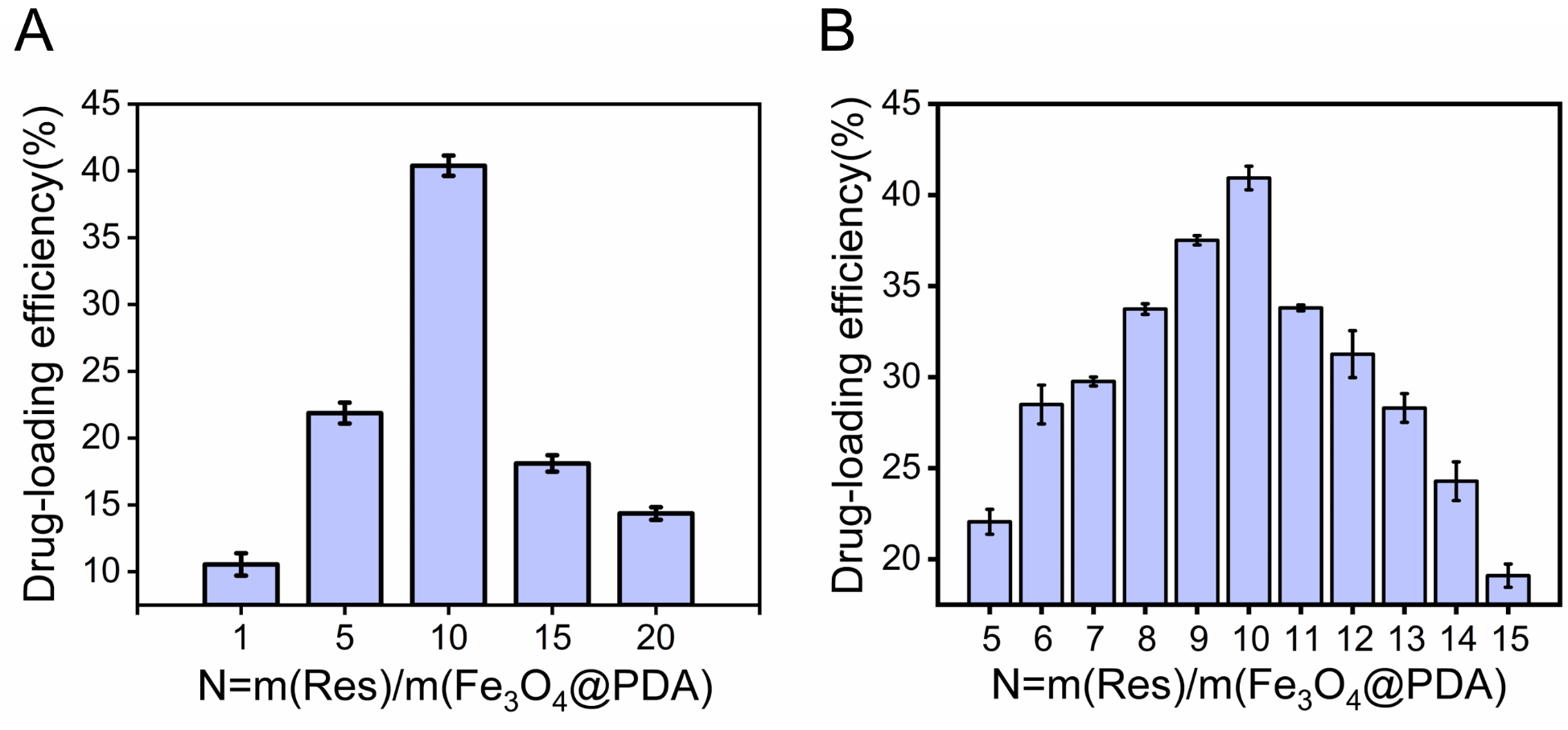


Figure S11. Drug loading efficiency at different mass ratios of drug and carrier (Fe_3_O_4_@PDA).

(A) Mass ratio from 1:1 to 1:20 and (B) Mass ratio from 1:5 to 1:15.


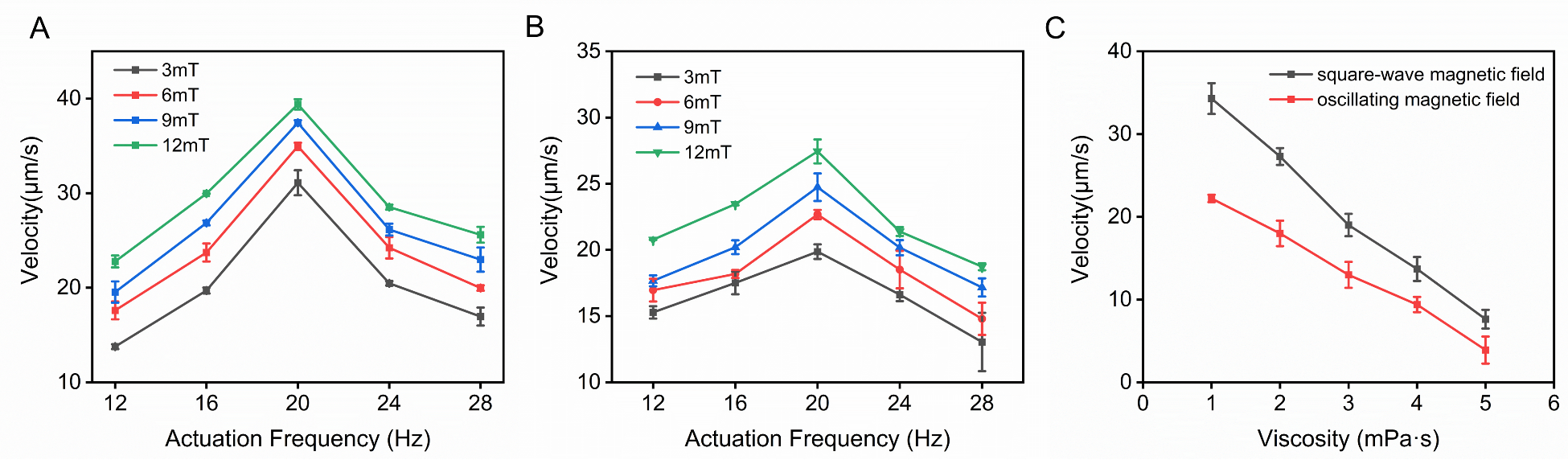


Figure S12. (A) Motion velocity of MNRs at different frequencies in a square wave oscillating magnetic field. (B) Motion velocity of MNRs at different frequencies in an oscillating magnetic field. (C) Motion velocity of MNRs in different magnetic fields and different viscosity environments.


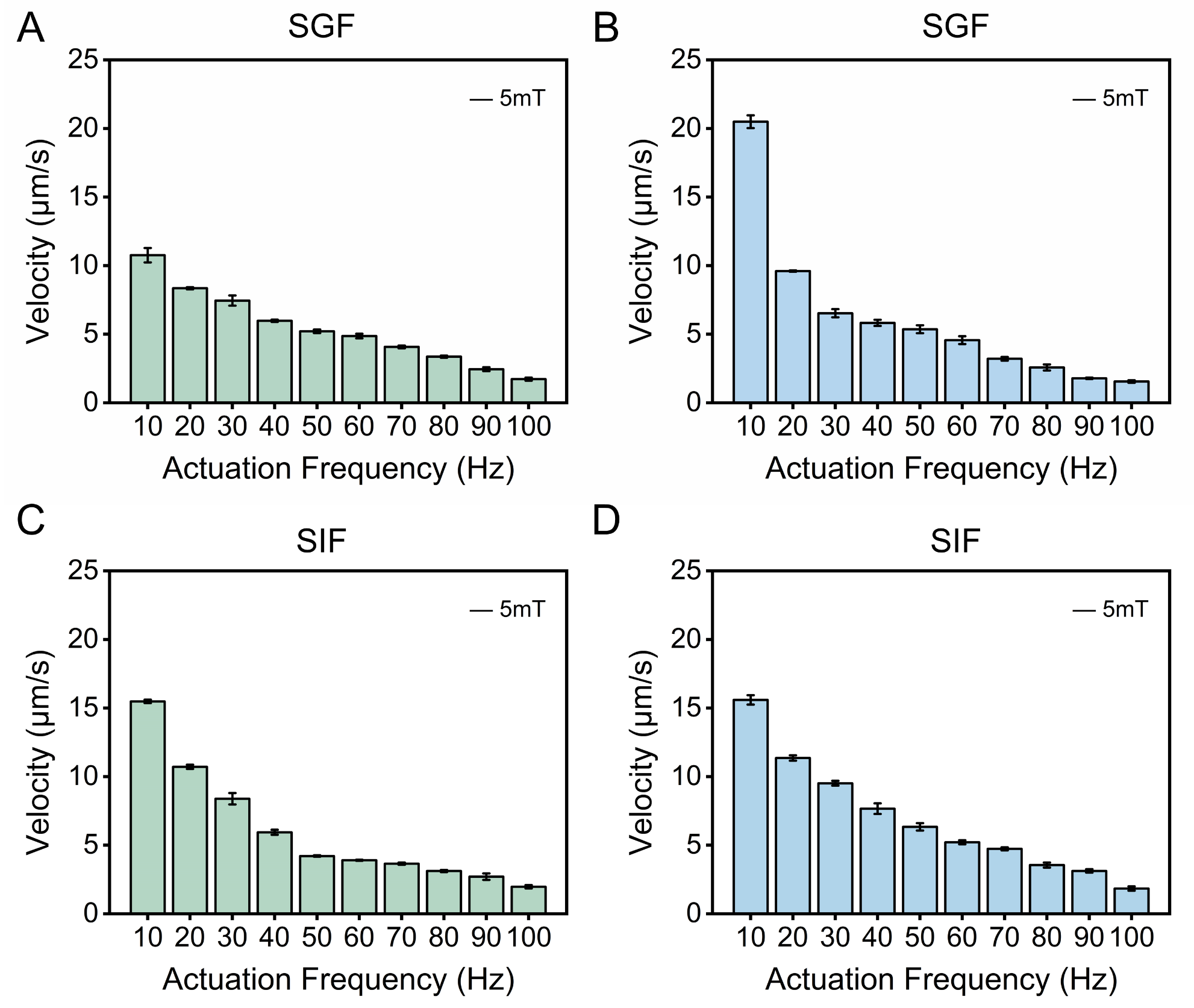


Figure S13. Average velocity versus frequency in square-wave magnetic field (A) and oscillating magnetic field (B) modes in SGF (n = 3; mean ± SD). Average velocity versus frequency in square-wave magnetic field (C) and oscillating magnetic field (D) modes in SIF (n = 3; mean ± SD). SGF: Simulated gastric fluid, SIF: Simulated intestinal fluid.


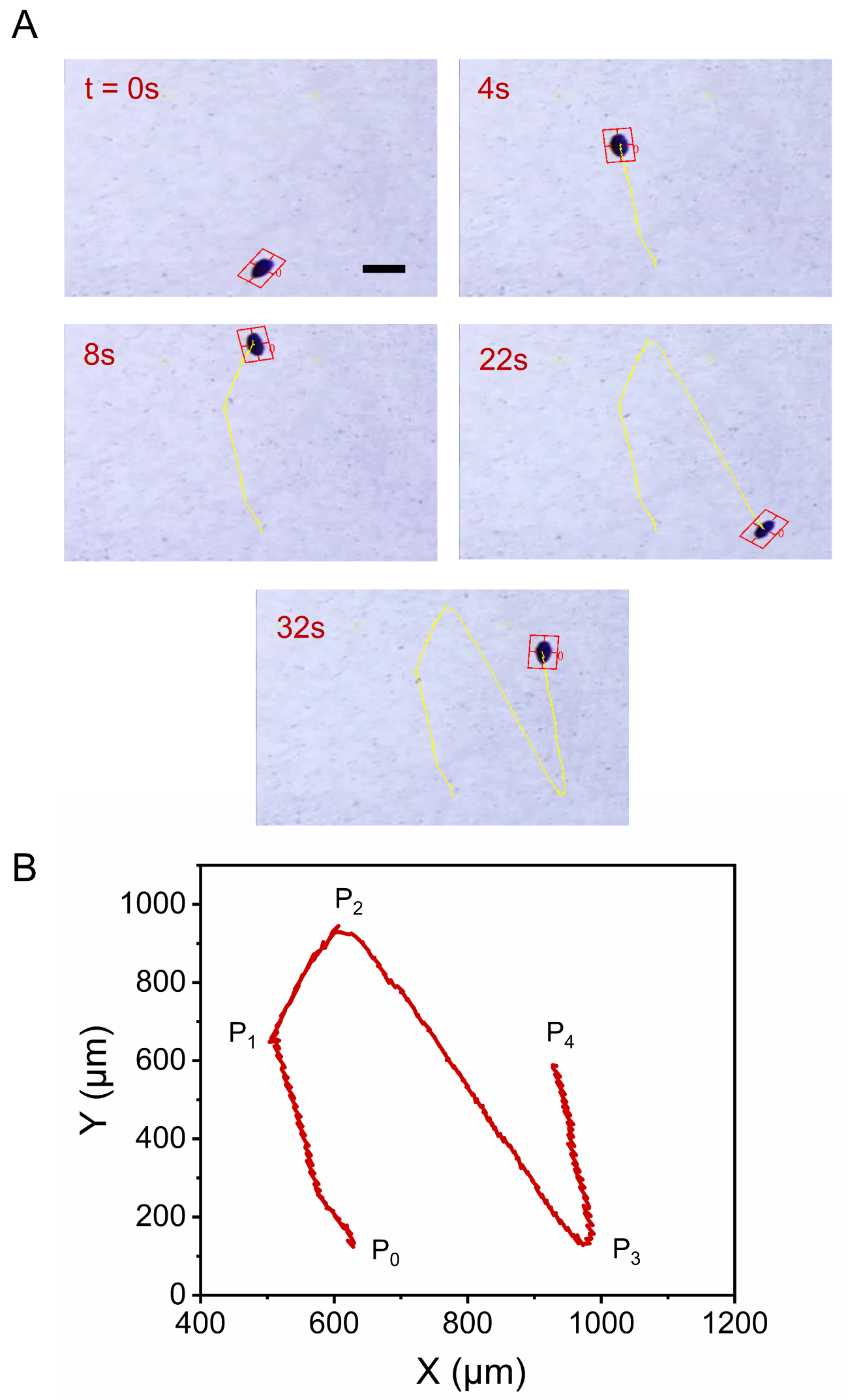


Figure S14. (A) Optical microscopy images and (B) motion of MNRs moving directionally to follow a “N” trajectory. Scale bar: 3mm.


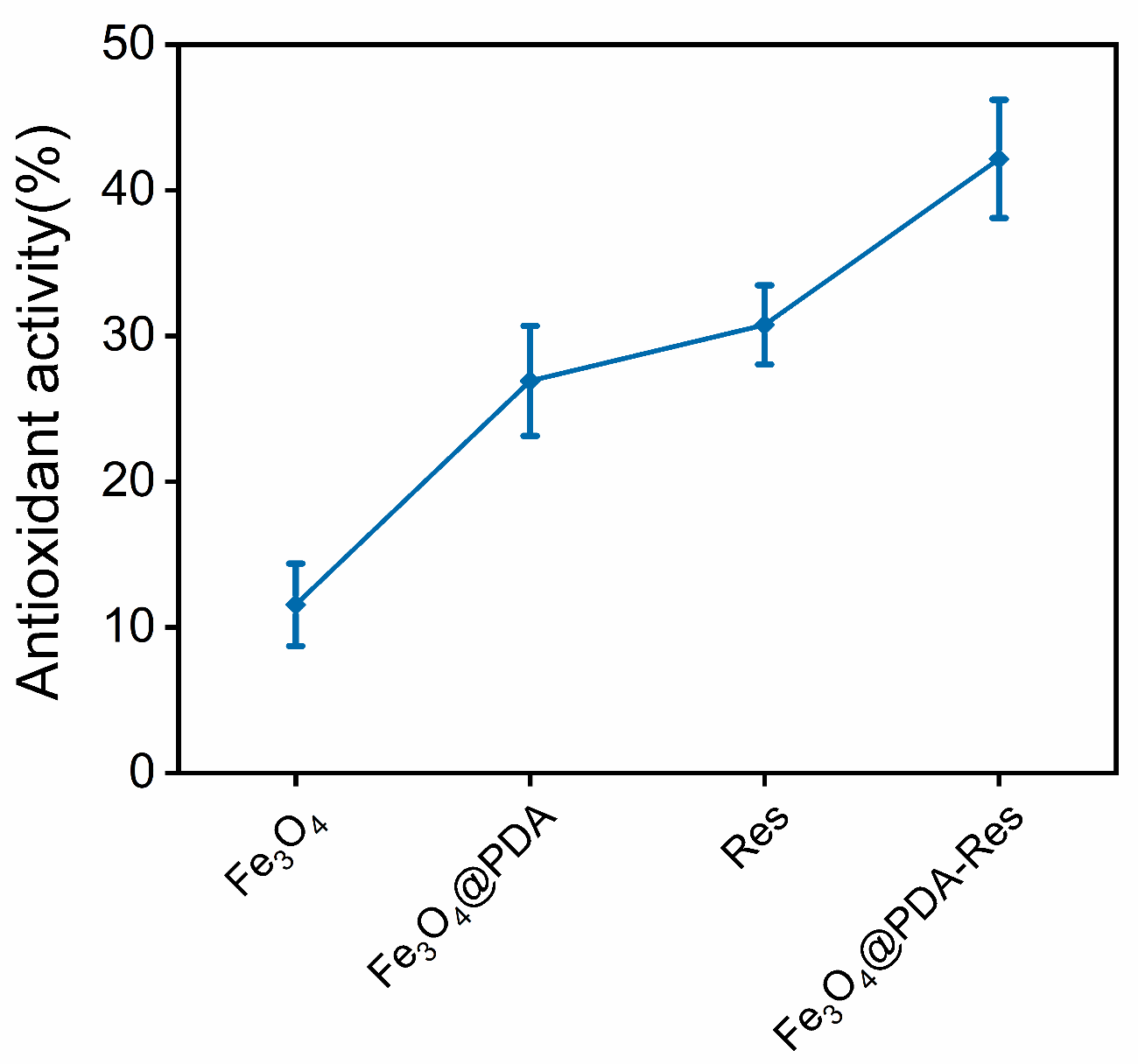


Figure S15. Antioxidant properties of MNRs and their components.


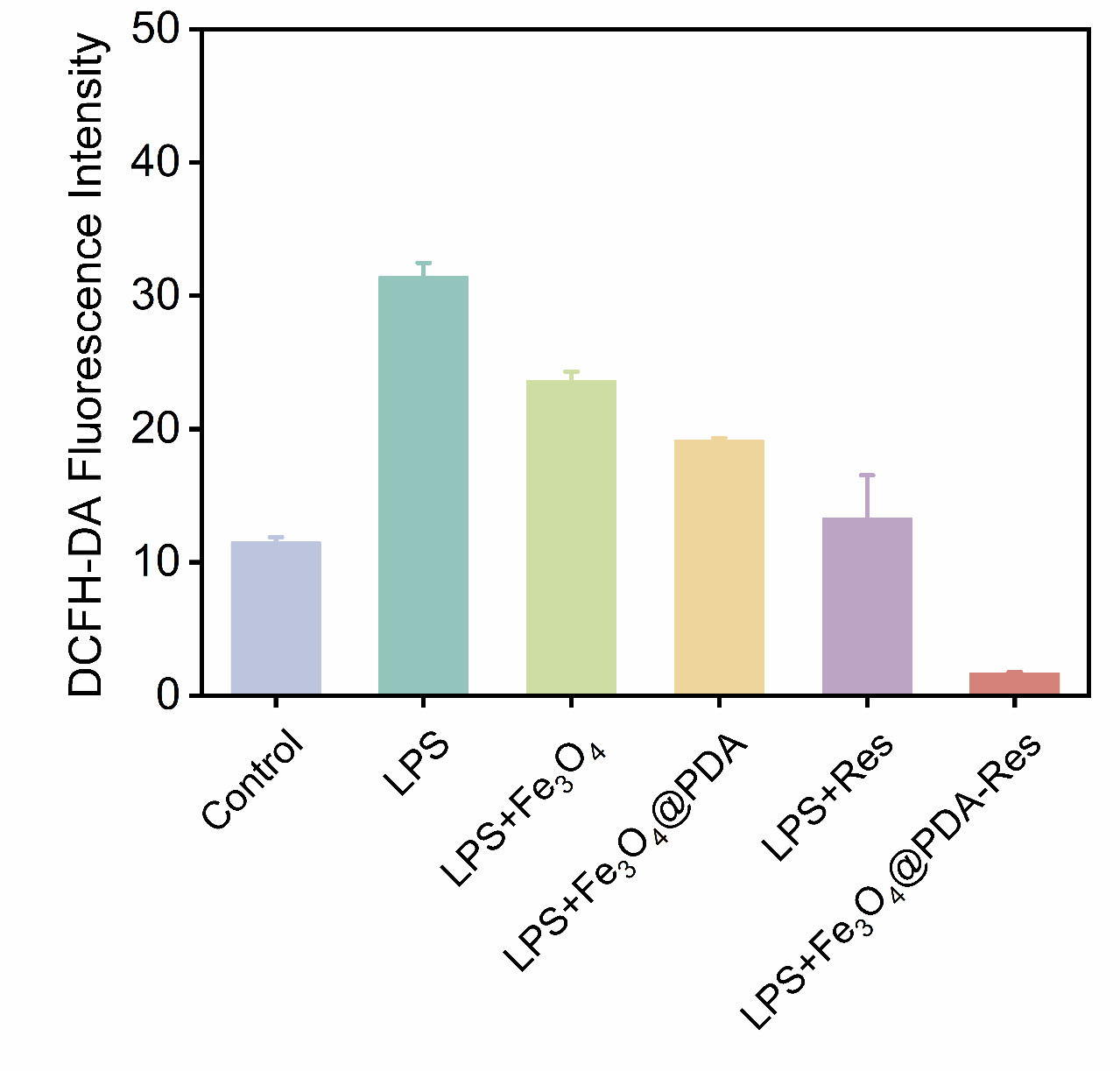


Figure S16. Representative ROS green fluorescence quantification of RAW264.7 cells after different treatments..

**

**

Figure S17. RAW264.7 cell viability of MNRs and their components.





Figure S18. Caco-2 cell viability of MNRs and their components.


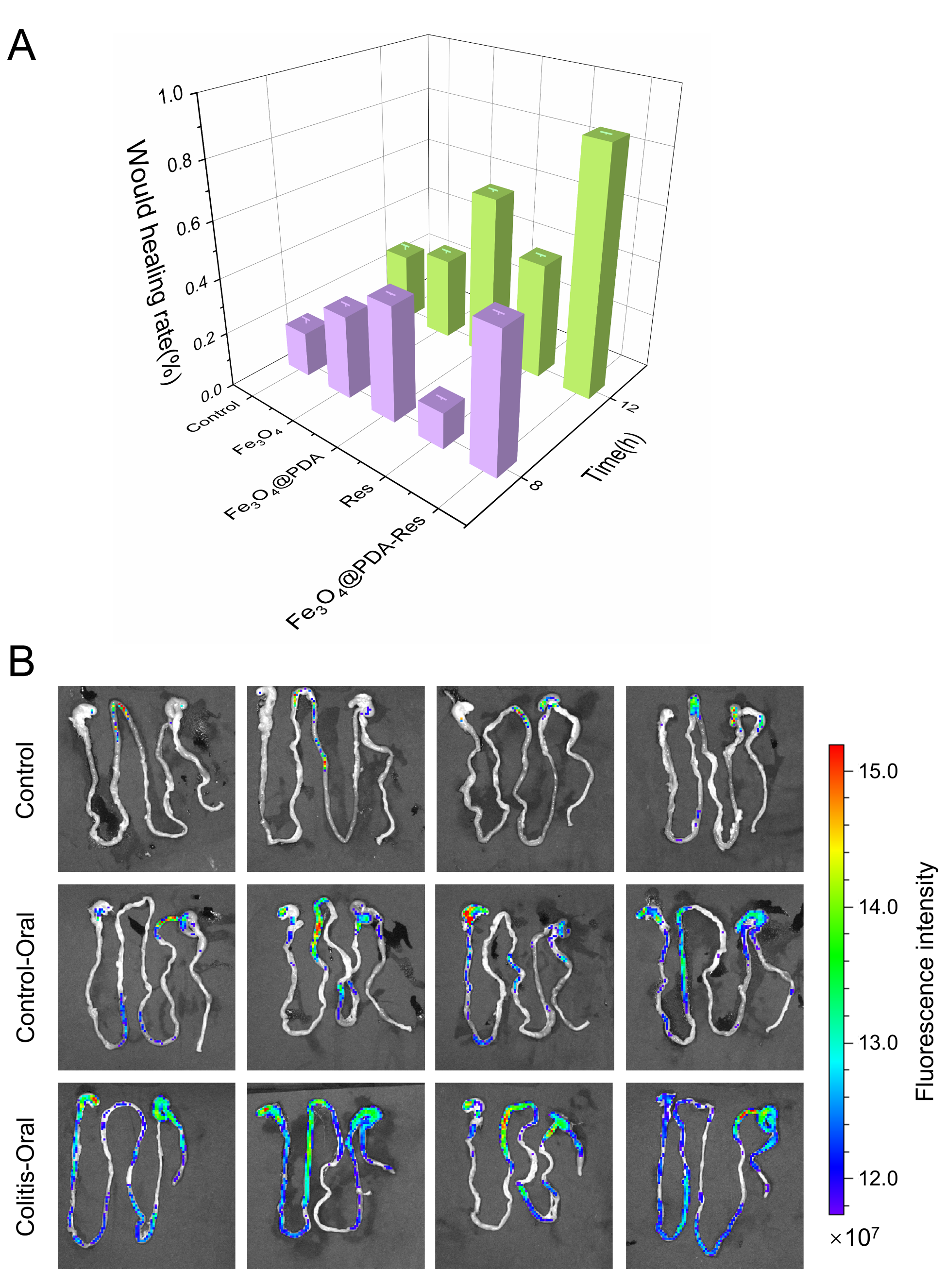


Figure S19. (A)Wound repair rate of Caco-2 cells after different treatments. (B) Fluorescence imaging of the GI tract, 10 hours after oral delivery of MNRs.


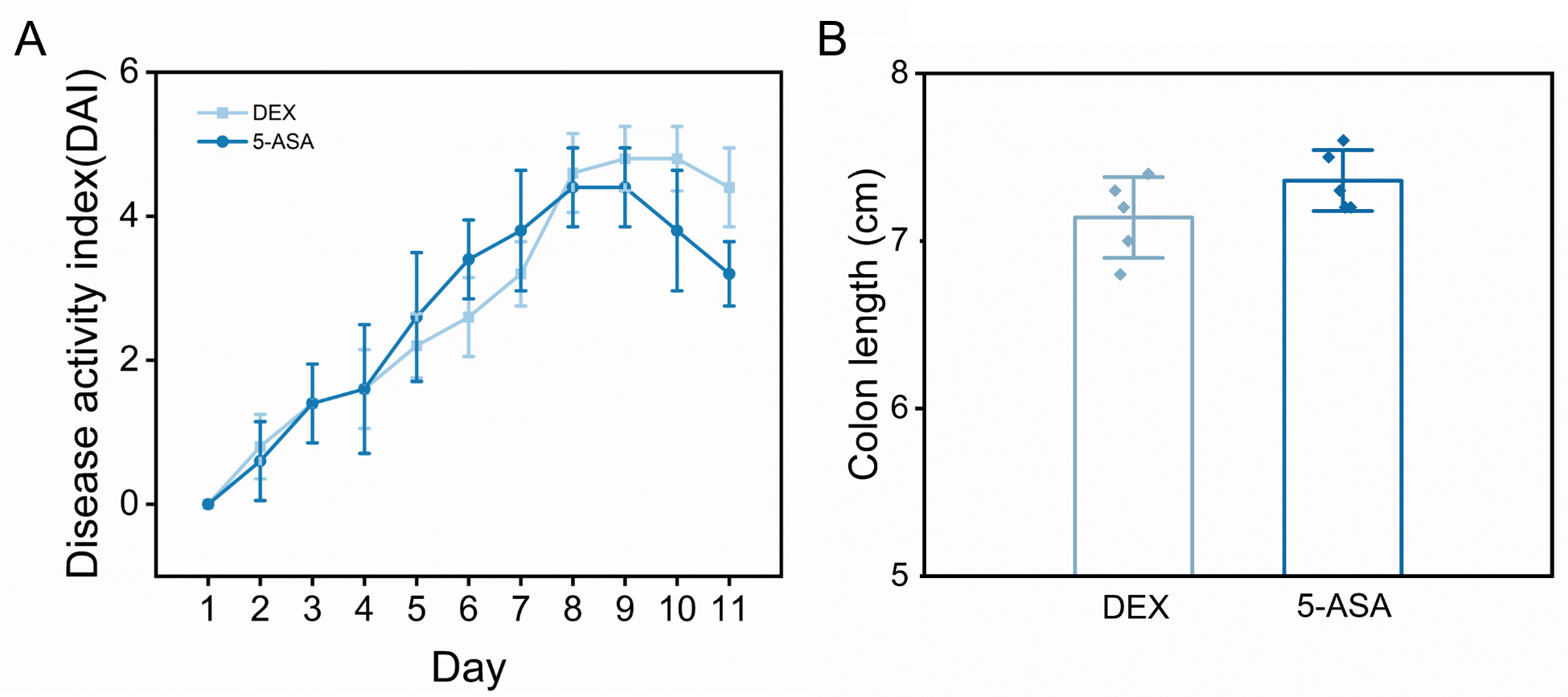


Figure S20. (A) DAI score values of the mice over 11 days. (B) Colon length in mice.


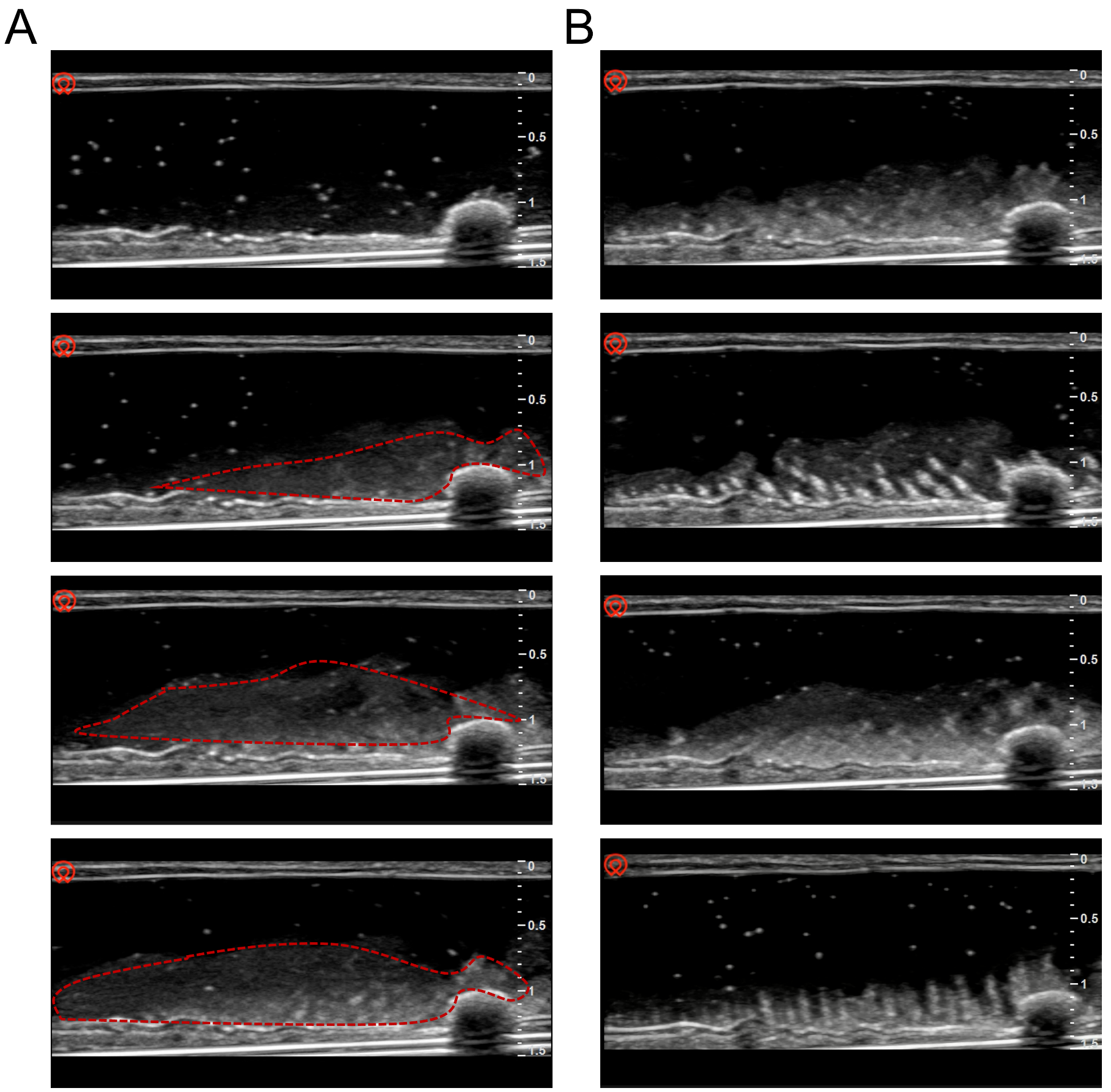


Figure S21. Isolated porcine intestinal tissue mimics real-time regulation in the mouse intestine. (A) Movement of MNRs in the intestine of isolated pigs (f=20Hz, 10mT). (B) Morphological transformations of MNRs in the isolated pig intestine (f=5Hz, 10mT).


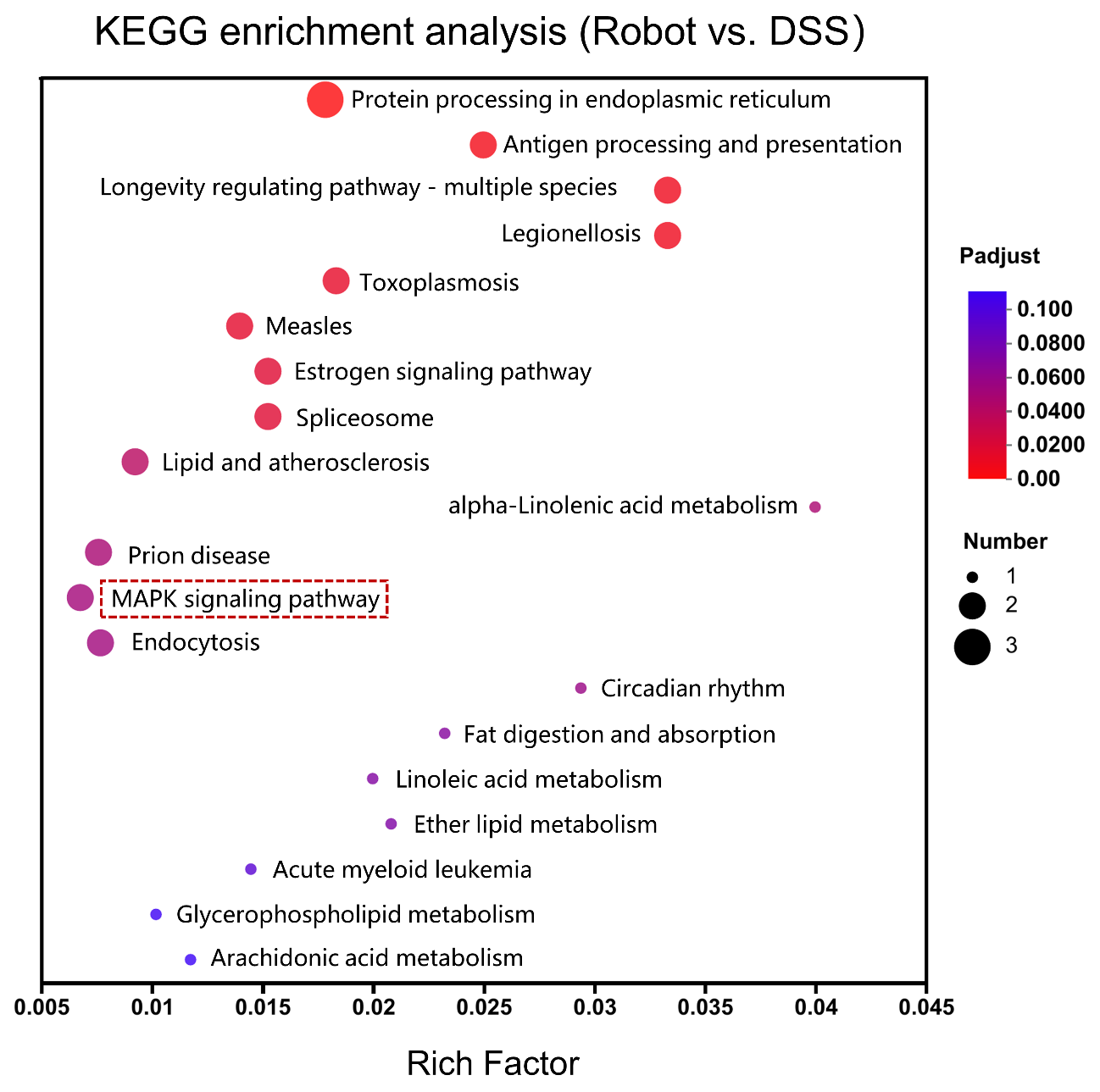


Figure S22. KEGG enrichment analysis of samples derived from DSS+Robot and DSS groups.


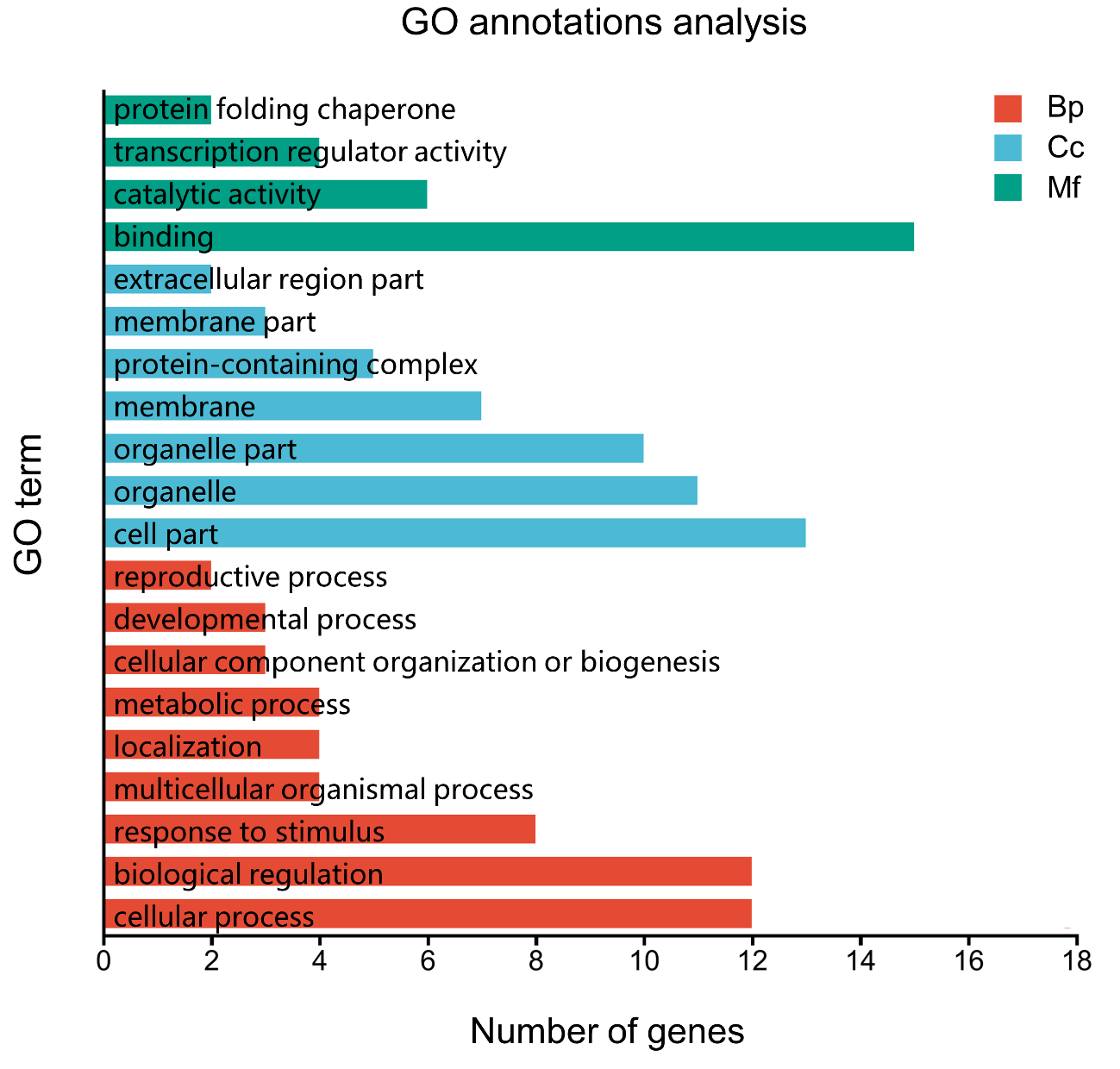


Figure S23. GO enrichment analysis of differentially expressed genes. Bp represents the “biological process”; Cc represents the “cellular component”; Mf represents the “molecular function”.


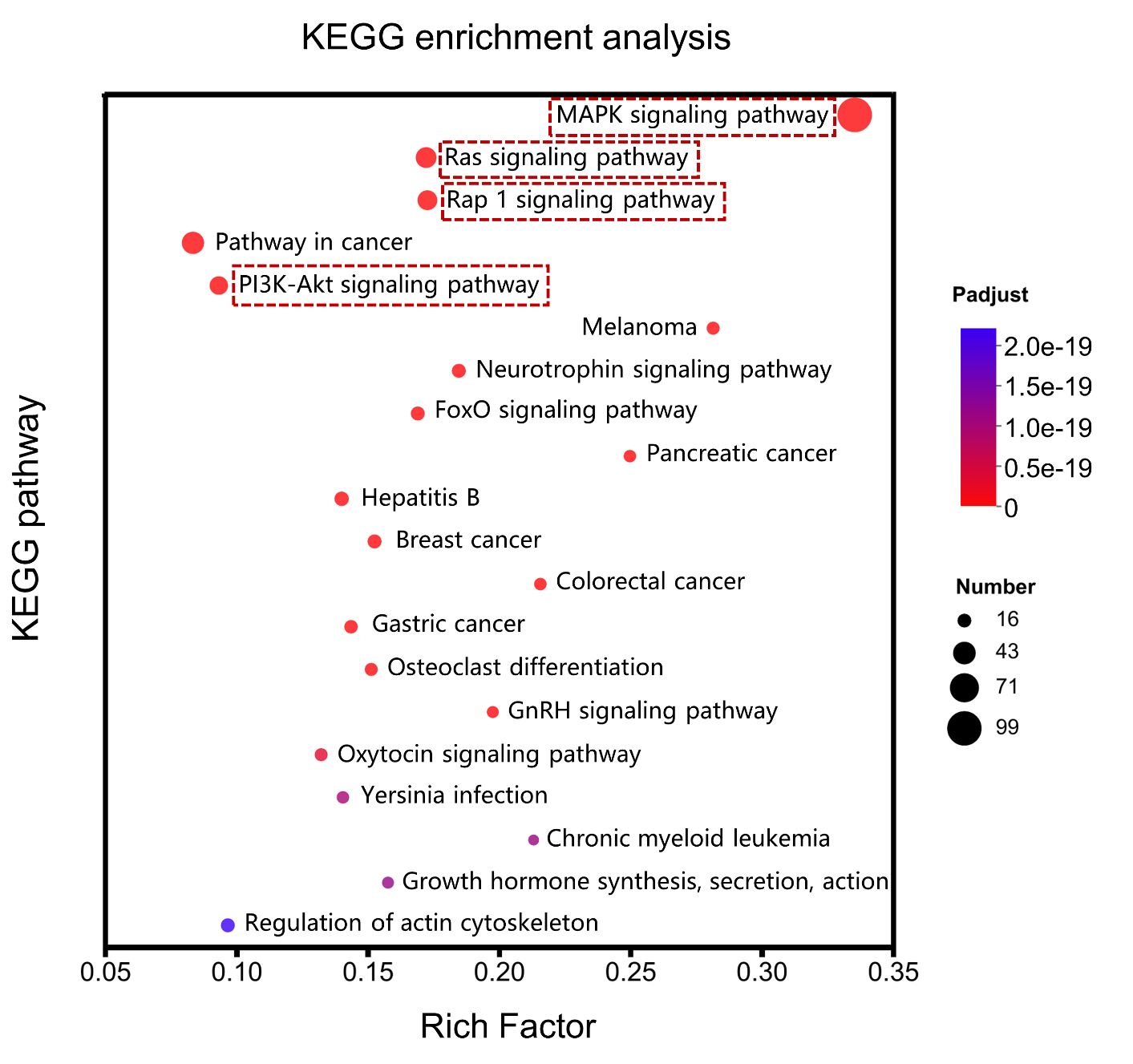


Figure S24. KEGG enrichment analysis of samples in ROS-related signaling pathway.


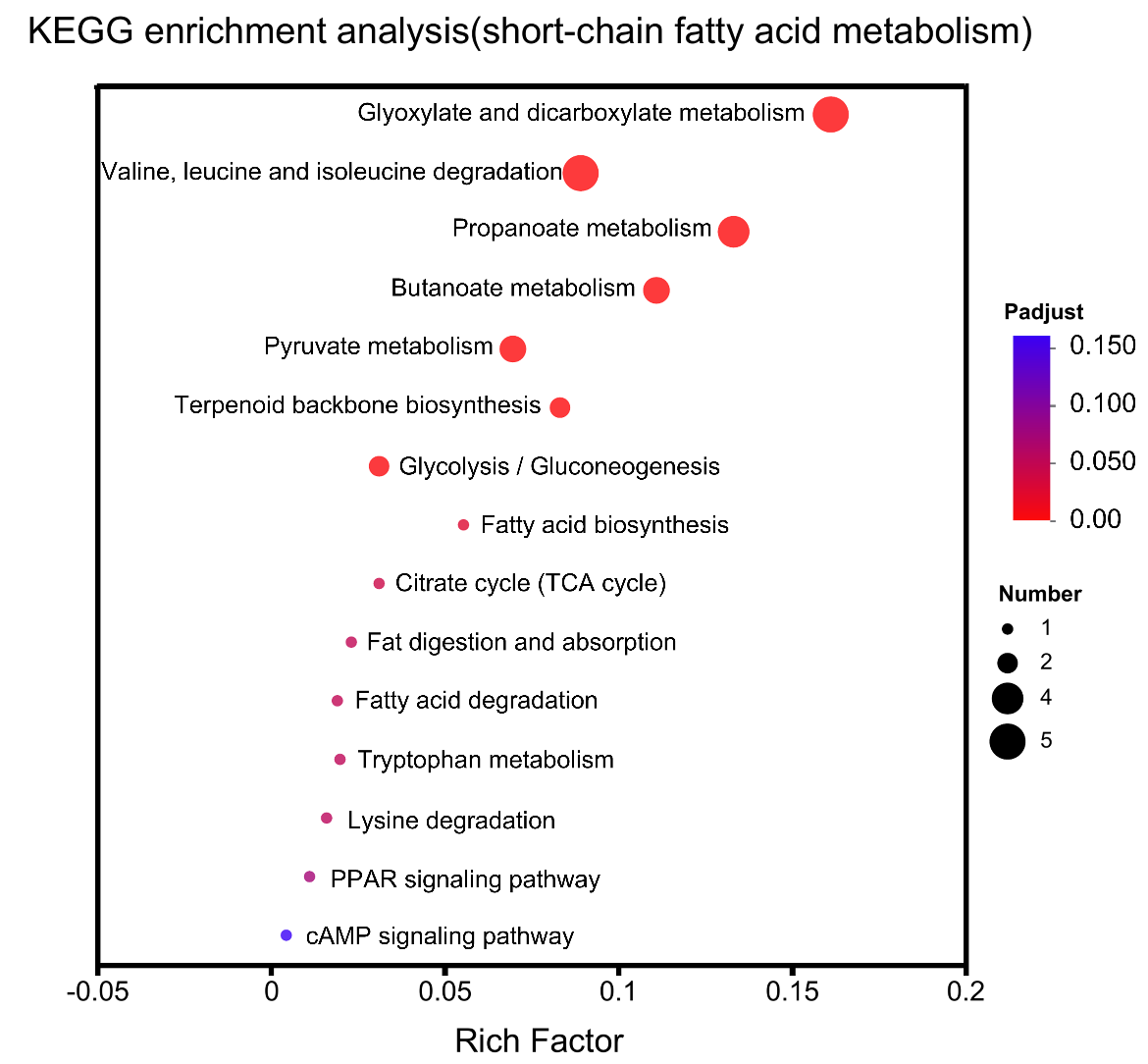


Figure S25. KEGG enrichment analysis of samples in short-chain fatty acid metabolism related pathway.


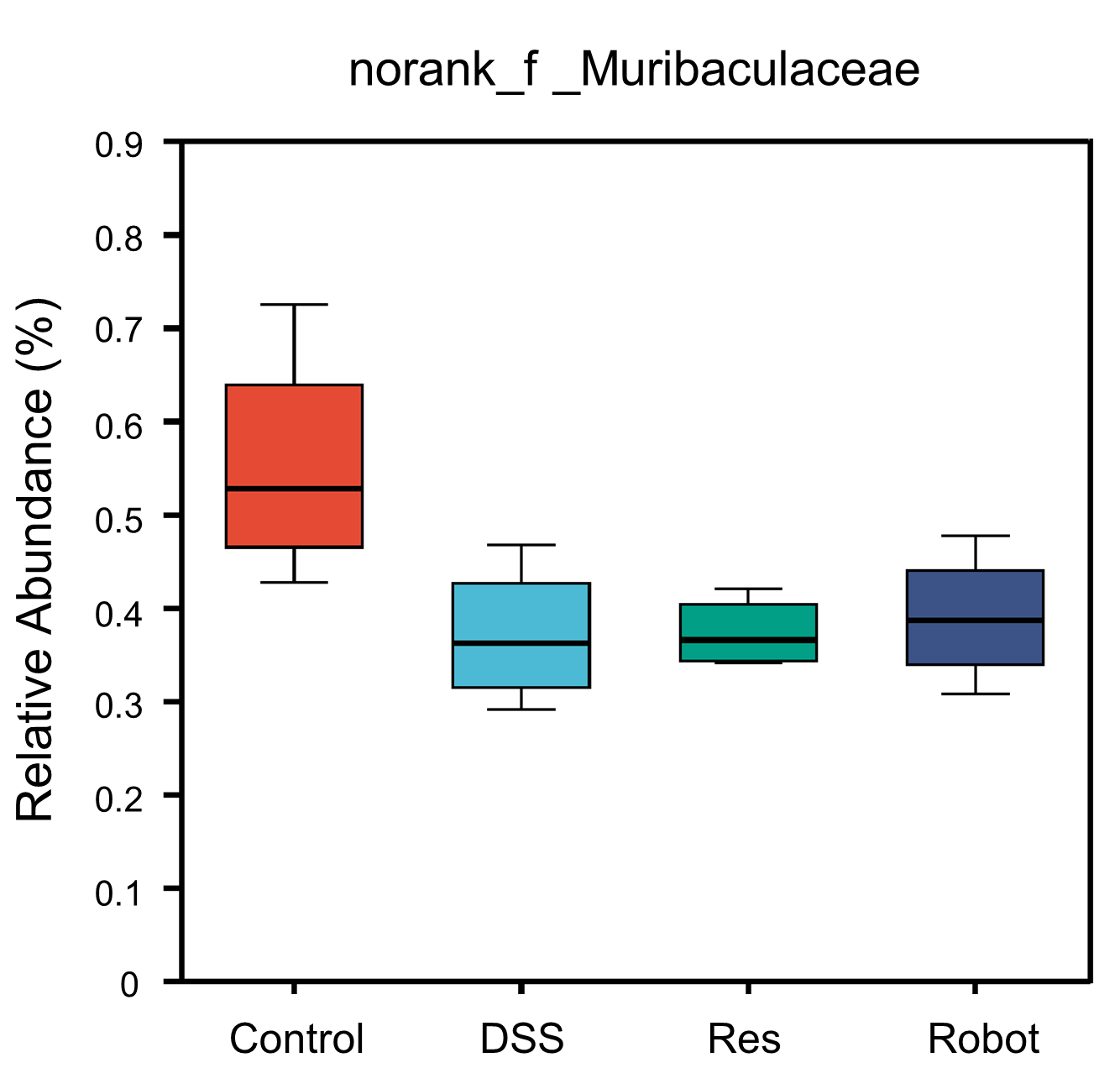


Figure S26. The relative abundance of norank_f_Muribaculaceae in each group of gut microbiota samples.


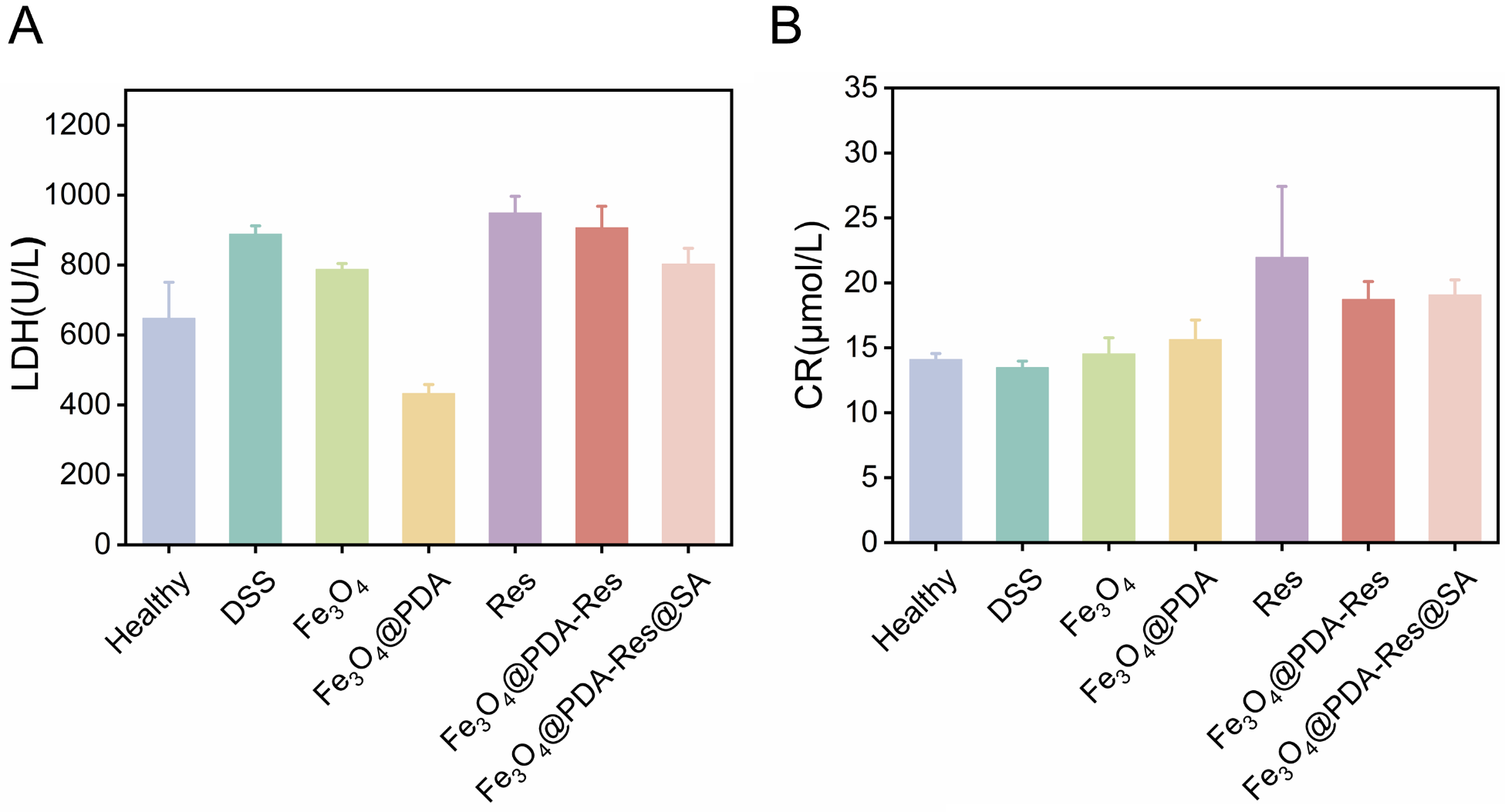


Figure S27. Comparison of (A) LDH (Lactate Dehydrogenase) and (B) CR (Creatinine) indicators of each group.


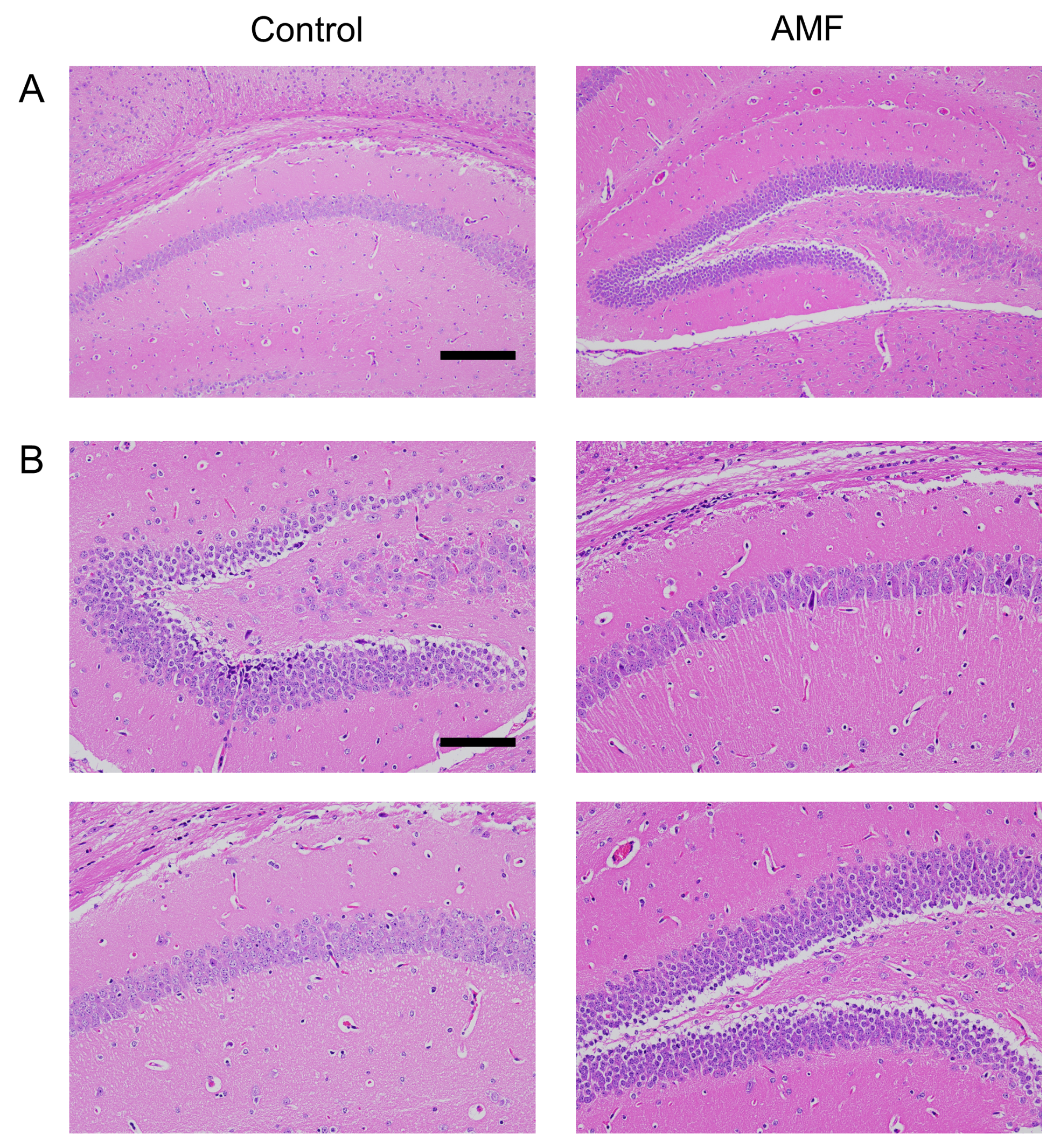


Figure S28. HE-stained images of mouse brain at different scales. Control group are untreated, healthy mice. AMF group are treated with an alternating magnetic field for 5 min. The scales for A and B are 200μm and 100μm respectively.

**
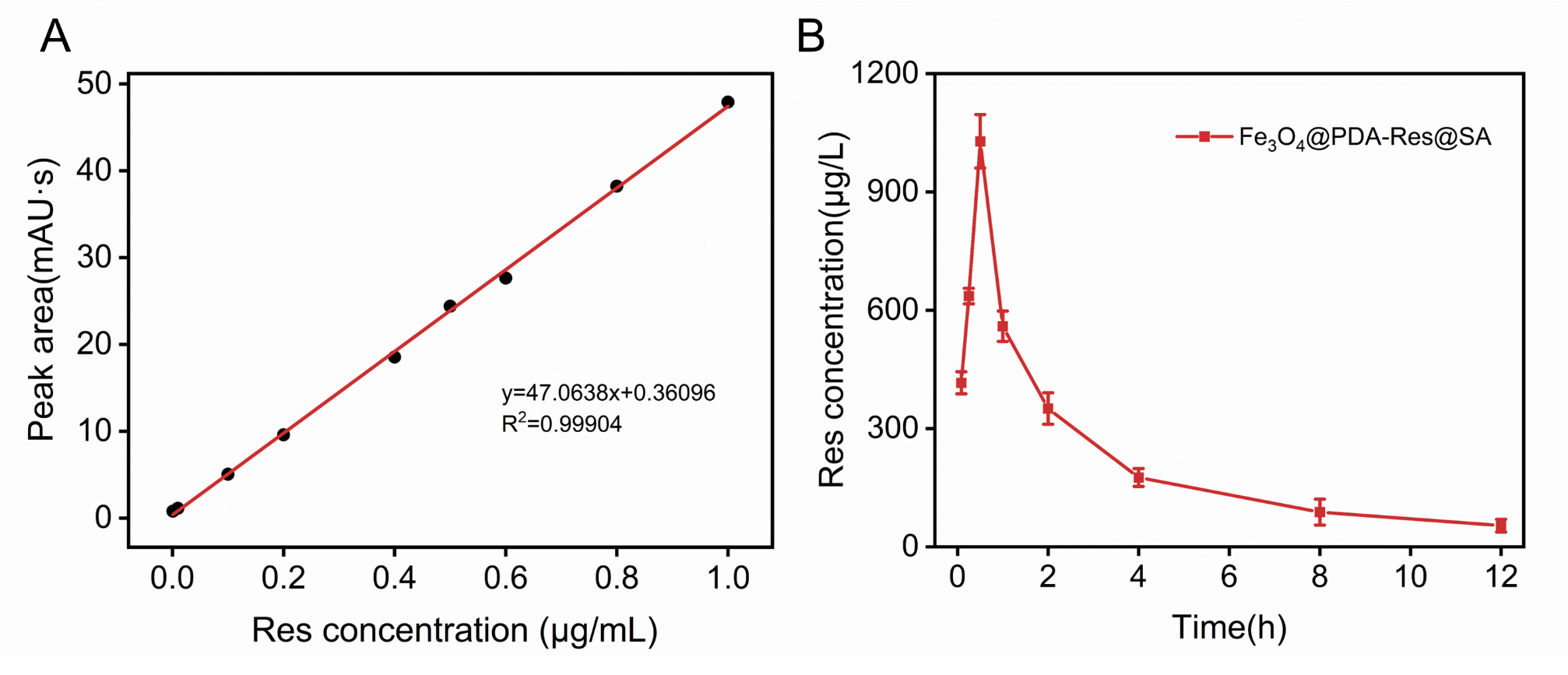
**

Figure S29. (A) Standard curve of resveratrol in mouse plasma. (B) Drug-time profile after gavage (n=5).





Figure S30. In vivo biodistribution of Fe over 3 days in mice. The iron concentrations in the organs were determined at different time points after injection using ICP-MS. Error bars indicate the SD for n = 3.

**
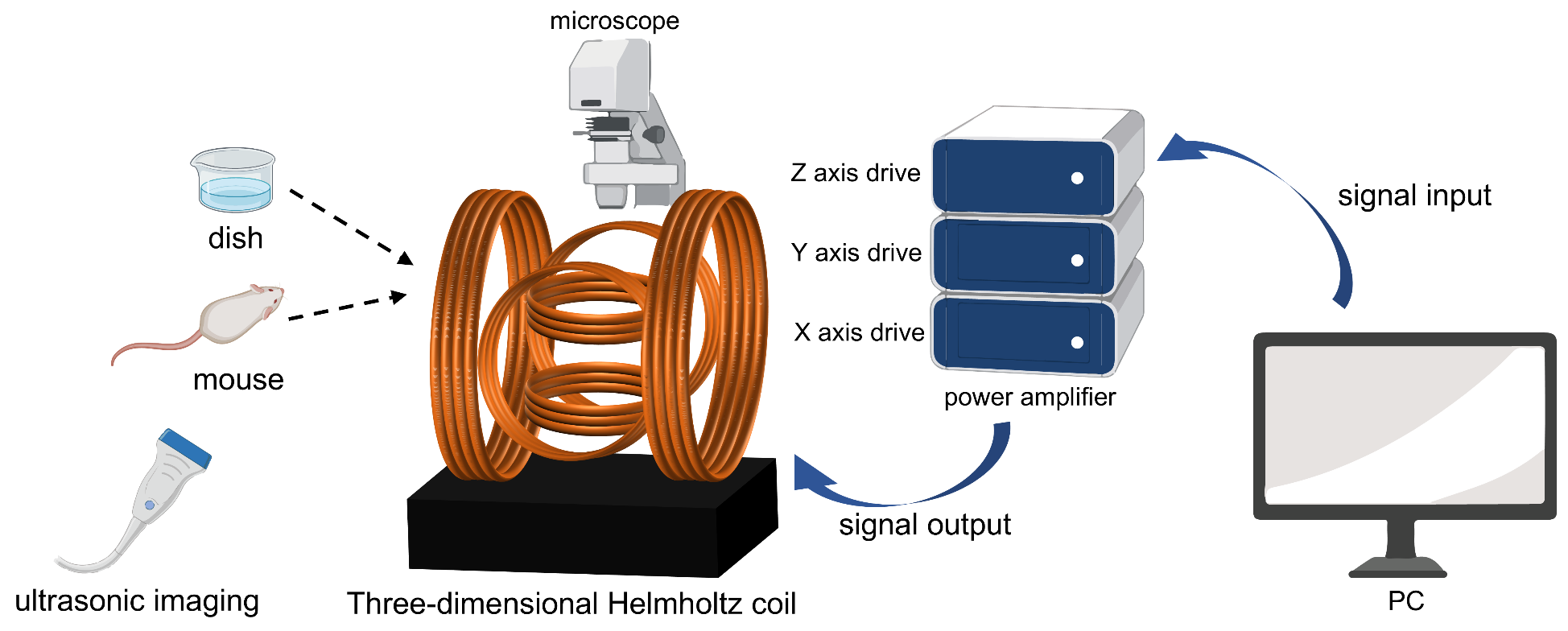
**

Figure S31. Schematic diagram of the magnetic field driven experimental setup.

**Table S1．**The comparison with similar magnetic driven MNRs.

| **Name** | **Motion speed**  **(****μm/s)** | **Application** | **Ref.** |
| --- | --- | --- | --- |
| Water-stable magnetic lipiodol micro-droplets | ≈ 4.6 | Drug delivery | [1] |
| Magnetic liquid-bodied microrobots | 35 | Overcoming biological barriers | [2] |
| Fe_3_O_4_-decorated multi-fluorescent magnetic microrobots | ≈ 8 | Monitoring intragastric acidity | [3] |
| Peanut-shaped magnetic microrobot | ≈ 25 | Reconfiguration into chain structures | [4] |
| Cas9-Engineered Magnetic Micromotors | 4.3 | Viral RNA assay | [5] |
| Vortex-shaped nanoparticle robot swarm | 35 | Hepatocellular carcinoma | [6] |
| Magnetite Micro/nanorobots | ≈ 39 | Inflammatory bowel disease | This work |

**Table S2．**Noncompartmental analysis parameters in mouse after oral administration of Fe_3_O_4_@PDA-Res@SA (n=5).

| **Parameters** | **Unit** | **Fe_3_O_4_@PDA-Res@SA** |
| --- | --- | --- |
| AUC_(0-t)_ | μg·h/L | 2475.897±109.543 |
| AUC_(0-∞)_ | μg·h/L | 2600.737±169.946 |
| MRT_(0-t)_  t_1/2_  T_max_  CL_z_**/**F  V_z_**/**F  C_max_ | h  h  h  L/h/kg  L/kg  μg/L | 3.192±0.224  2.675±0.401  0.5  19.291±1.257  74.053±7.858  1028.281±67.772 |

Movie S1. Oscillatory motion in an oscillating magnetic field. Scale bar is 1 mm.

Movie S2. MNRs undergo morphological changes and move in different directions. Scale bar is 3 mm.

Movie S3. The direction control of MNRs. Scale bar is 1 mm.

Movie S4. Trajectory "N" of the MNRs. Scale bar is 800 μm.

Movie S5. Trajectory "C" of the MNRs. Scale bar is 1 mm.

Movie S6. Trajectory "U" of the MNRs. Scale bar is 1 mm.

Movie S7. Trajectory "G" of the MNRs. Scale bar is 1 mm.

Movie S8. Movement of MNRs in the intestine of isolated pigs.

Movie S9. Morphological transformations of MNRs in the isolated pig intestine.

Movie S10. MNRs are in full contact with the intestinal wall in the narrowed isolated pig intestine.

**Reference**

[1] E. Ren, J. Hu, Z. Mei, L. Lin, Q. Zhang, P. He, J. Wang, T. Sheng, H. Chen, H. Cheng, T. Xu, S. Pang, Y. Zhang, Q. Dai, X. Gao, H. Liu, H. Li, Y. Zhao, Z. Gu, X. Yan, G. Liu, *Adv. Mater.* **2025**, *37*, 2412187.

[2] X. Wu, L. Zhang, Y. Tong, L. Ren, H. Guo, Y. Miao, X. Xu, Y. Ji, F. Mou, Y. Cheng, J. Guan, *ACS Nano*. **2024**, *18*, 29558.

[3] N. Senthilnathan, C.M. Oral, A. Novobilsky, M. Pumera, *Adv. Funct. Mater.* **2024**, *34*, 2401463.

[4] H. Xie, M. Sun, X. Fan, Z. Lin, W. Chen, L. Wang, L. Dong, Q. He, *Sci. Adv.* **2019**, *4*, eaav8006.

[5] Y. Song, H. Park, P. Thirumalaraju, N. Kovilakath, J.M. Hardie, A. Bigdeli, Y. Bai, S. Chang, J. Yoo, M.K. Kanakasabapathy, S. Kim, J. Chun, H. Chen, J.Z. Li, A.M. Tsibris, D.R. Kuritzkes, H. Shafiee, *ACS Nano*. **2025**, *19*, 8646.

[6] L. Jia, Y. Dai, Y. Xu, H. Sun, H. Gao, H. Hao, L. Wang, J. Xu, J. Shang, G. Li, Y. Xu, L. Feng, *Small*. **2025**, *21*, 2402909.
